# Supplementary material for: Targeting ocular tissues with intravenously administered aptamers selected by in vivo SELEX
Source: Mol Ther Nucleic Acids. 2024 Oct 4;35(4):102352. doi: 10.1016/j.omtn.2024.102352 (PMC11513532; doi:10.1016/j.omtn.2024.102352)
Supplement: Document S1. Figures S1–S3, Tables S1, and S2 [file mmc1.pdf]

OMTN, Volume *35*

Supplemental information

Targeting ocular tissues with intravenously administered aptamers selected by *in vivo* SELEX

Sonja Korhonen, Katja Stenberg, Umair Seemab, Piia Bartos, Katariina Mäkiniemi, Jørgen Kjems, Daniel Miotto Dupont, and Astrid Subrizi

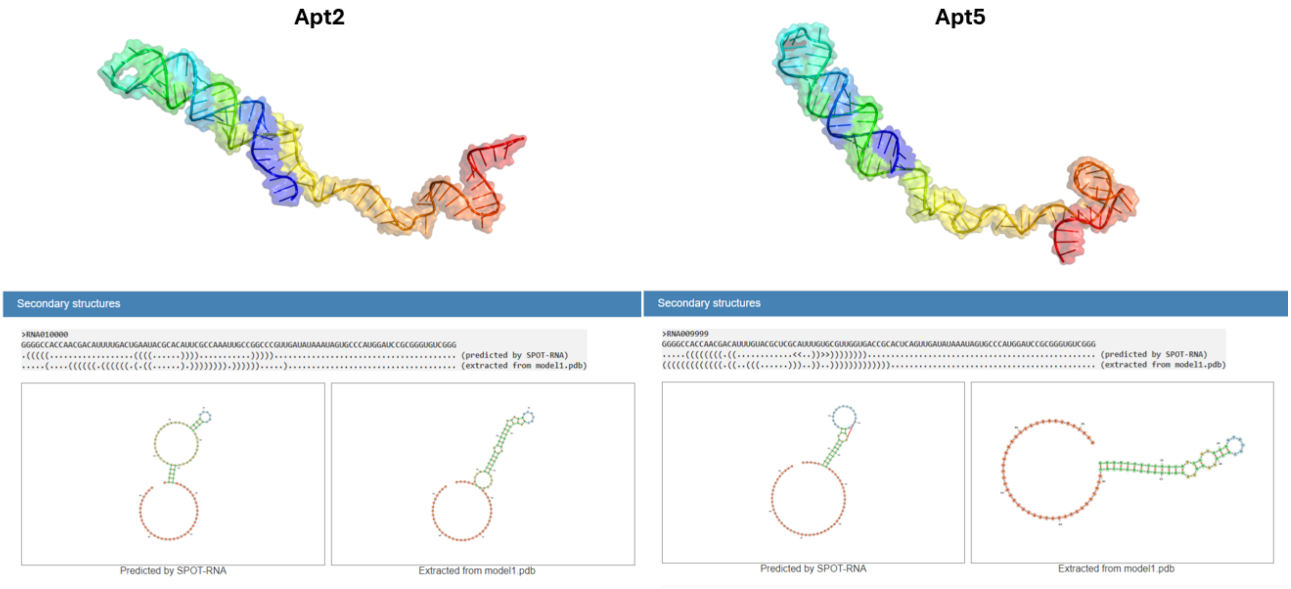

Figure S1: Secondary structures and 3D models for Apt2 and Apt5 built based on de novo folding guided by deep learning restraints with the trRosettaRNA.

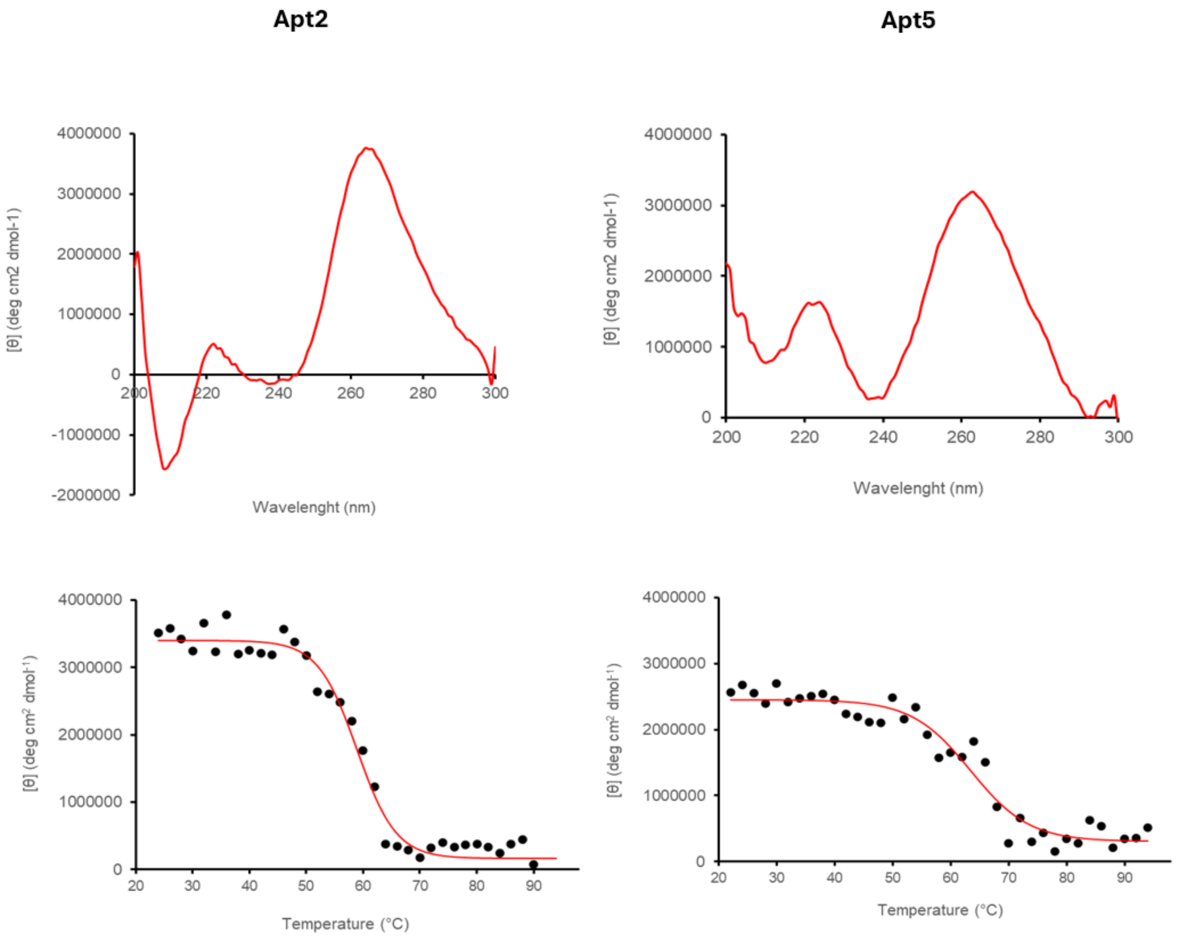

Figure S2. Characterization of the Aptamers Apt2 and Apt5 using circular dichroism (CD) and melting temperature analysis. The final spectra were represented as a unit of molar ellipticity. The top row represents the smoothed CD spectra of Apt2 and Apt5 and the lower row melting curves used for melting temperature detection.

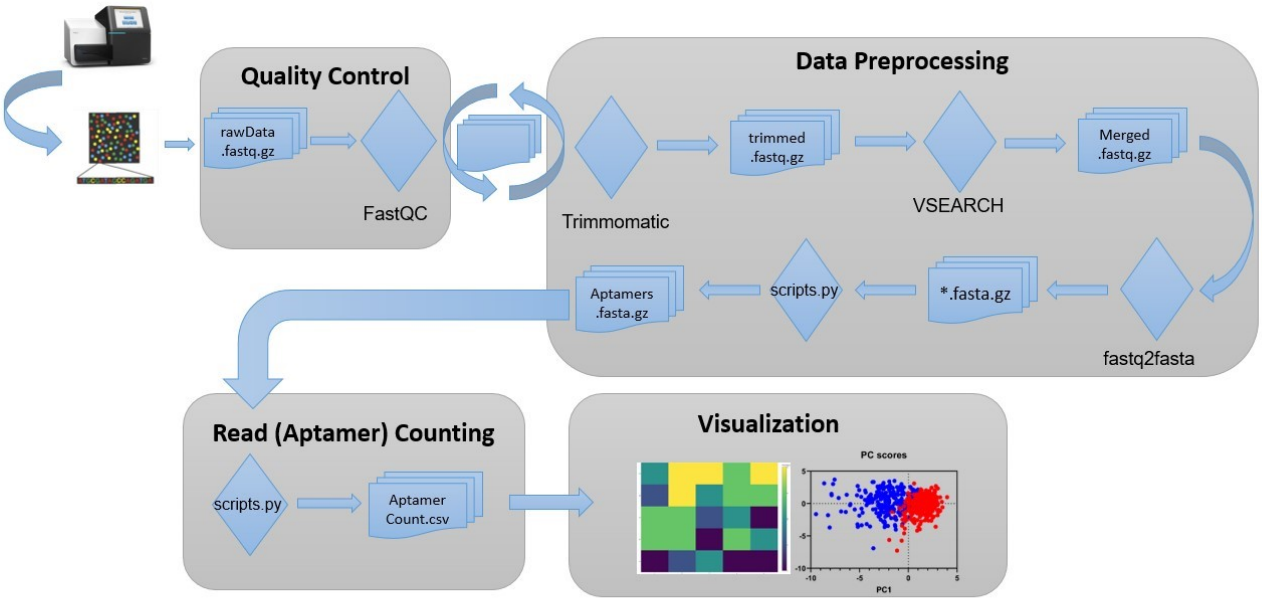

Figure S3: Aptamer data analysis workflow from raw data files to read count in each sample for further downstream analysis.

Table S1: Percentage of the 50 most enriched 36 nucleotide sequences of total sequence count pooled from SELEX cycles 10, 12, and 14. Only the 36 nucleotide-long variable regions are shown.

Aptamer sequence	Percentage of the pool (%)
GATTGTCCCAAATTATCCTTAGAACTTTTACCTCCA	34.53
TTGACTGAATACGCACATTCGCCAAATTGCCGGCCC	3.89
ATGCCGTCCGACCCACTCGTACGGCACTATCTCCCC	3.48
CAATGCCCTCATGTTTTTGCTCAAAACATCACTGCT	2.33
TGTACGCTCGCATTTGTGCGTTGGTGACCGCACTCA	0.73
GATTGTCCCAAATTATCCTTAGAACTTTTATCTCCA	0.67
TCTGCTAGGCGGGATTTTCGTCAGTGTCCCACCCAT	0.54
ACAAACCAAGAGTCGCTATTGTCGGTTGATGCTCCC	0.54
GGTAGCACCCGCCAATCGACTTTTCACCGCAGAAGC	0.53
ATGCCGTCCGACCCACTCGTACGGCACTAACTCCCC	0.43
GATTGTCCCAAACTATCCTTAGAACTTTTACCTCCA	0.28
GTTGCCCTTCAAACTGACGACCGACCGTCGTGAAAA	0.24
GGTGTCCTGCCATATAACTAGTTACCTCGACCTATC	0.23
GTCGCACCATCGAGCGCGCACAATAGCGTGTCCCCC	0.22
GATTGCCCCAAATTATCCTTAGAACTTTTACCTCCA	0.19

GGATGGGCTCCTTCGCAACGATGTACCGCATACGAC	0.19
ATGCCGTCCGATCCACTCGTACGGCACTATCTCCCC	0.16
GGTTCGTACCCTCTATGACATCACGGCTCGCGCGAT	0.14
CTACTCAACCGGTTTGATCTAGATTGCTCGGCCCCA	0.14
GTTGCTGACCAGTTGCGTTACGACATCACTCTCGAT	0.14
GATCGTCCCAAATTATCCTTAGAACTTTTACCTCCA	0.14
GAATGAACATCCAGTCTTTGTCTTTCCTTGCTCCCC	0.13
TTTCGCAGTCGATATCTTCTTTCGTTGGCACCCCAA	0.13
GGTTTCGCCAATCACTACAACGTACGTGCCCGTATG	0.13
CCGGTGAGCCTTTCCGCACGCTTGAGCAAGTCACGC	0.13
TGTTGTCTGGATTTGCCGATAGTCACGTTACGTAAC	0.12
ACAATGTCACTTAAATCCCGGCCCGAATCACGTAAC	0.12
CGTATGTCTGACCCTGACATAAAACGTTAGCGTAAC	0.12
TGGTTAAGAGCAACTGCCCTAATCTCACAATGCCCC	0.11
GGTTGCCTGCATTTACGCTGTTTACGCCTTCGCAGC	0.11
CGTGCCGACCCGCATGTCCACGGTGCTCCACACACC	0.11
GGTCTGCCTTTTCATATAACCGTTTATCTCACTCCC	0.11
GATTGTCCCAAATCATCCTTAGAACTTTTACCTCCA	0.10
TACTTACGTTTATGTCCTGCGGTCTACGCCCTCCCC	0.10
GTTTGGCTTCCATCCGCGTATCAACCGTCGACCCGT	0.10
CGCGCTCGTTAGCAATTTCGGTGTCCCGTTATAATC	0.10
GGCGAGCCGCAGCACCGACTTCACGCATACGCGTTC	0.10
CTCGGAAAACCGCTGATTTCGCTCACTGACCCCACA	0.09
GTTACAAAATGTCCCCGCATGCGTCCACGCTCTCGC	0.09
GAGTTGCCCTTATCCTCCCAACGCATGCACCAGCGC	0.08
GGAATGCCCGCCTCTAACGCTCAGGCCGTCTGCTGC	0.08
CACCCCAACGCCCCTTGCGGACAAGCGTTGTCCTGC	0.08
TGTTCCCTTATTGTAAACACACGTGCCCTCGTGTGC	0.08
CGCCCATATCACTGGGACTTGACGTATCTTGGCCGC	0.08
CGGCTGCCAACCGCATATCGTGAAGGCTGGCTTCGA	0.08
GATTGTCCCAAATTATCCTTAGAACCTTTACCTCCA	0.08
TCTTGCCGAGTGCGCATCGACAAGTCAGAATGCCCC	0.07
GATTGTCCCAAATTATCCTTAGAACTTTTACCTCAA	0.07
CGTACAGACGTGCACGCCAAACGTCGTACGGCCTCG	0.07
CGCACGCAACGCCGCTGTTCCGTGAGATTGAGCCCT	0.07

Table S2: Change in the percentage of the 50 most enriched 36 nucleotide sequences of total sequence count from SELEX cycles 10, 12, and 14. Only the 36 nucleotide-long variable regions are shown.

Aptamer sequence	Percentage of the pool (%)		
	Cycle 10	Cycle 12	Cycle 14

GATTGTCCCAAATTATCCTTAGAACTTTTACCTCCA	26,10	40,05	38,83
TTGACTGAATACGCACATTCGCCAAATTGCCGGCCC	1,31	4,16	5,27
ATGCCGTCCGACCCACTCGTACGGCACTATCTCCCC	4,70	5,12	2,39
CAATGCCCTCATGTTTTTGCTCAAAACATCACTGCT	2,40	3,78	1,67
TGTACGCTCGCATTTGTGCGTTGGTGACCGCACTCA	0,03	0,10	1,51
GATTGTCCCAAATTATCCTTAGAACTTTTATCTCCA	0,29	0,74	0,86
TCTGCTAGGCGGGATTTTCGTCAGTGTCCCACCCAT	0,02	0,22	1,03
ACAAACCAAGAGTCGCTATTGTCGGTTGATGCTCCC	0,39	0,58	0,65
GGTAGCACCCGCCAATCGACTTTTCACCGCAGAAGC	0,52	0,73	0,46
ATGCCGTCCGACCCACTCGTACGGCACTAACTCCCC	0,14	0,41	0,62
GATTGTCCCAAACTATCCTTAGAACTTTTACCTCCA	0,16	0,30	0,35
GTTGCCCTTCAAACTGACGACCGACCGTCGTGAAAA	0,15	0,29	0,26
GGTGTCCTGCCATATAACTAGTTACCTCGACCTATC	0,01	0,06	0,46
GTCGCACCATCGAGCGCGCACAATAGCGTGTCCCCC	0,06	0,15	0,36
GATTGCCCCAAATTATCCTTAGAACTTTTACCTCCA	0,15	0,24	0,21
GGATGGGCTCCTTCGCAACGATGTACCGCATACGAC	0,26	0,25	0,14
ATGCCGTCCGATCCACTCGTACGGCACTATCTCCCC	0,21	0,25	0,11
GGTTCGTACCCTCTATGACATCACGGCTCGCGCGAT	0,29	0,17	0,08
CTACTCAACCGGTTTGATCTAGATTGCTCGGCCCCA	0,16	0,23	0,10
GTTGCTGACCAGTTGCGTTACGACATCACTCTCGAT	0,16	0,07	0,20
GATCGTCCCAAATTATCCTTAGAACTTTTACCTCCA	0,07	0,16	0,18
GAATGAACATCCAGTCTTTGTCTTTCCTTGCTCCCC	0,16	0,16	0,13
TTTCGCAGTCGATATCTTCTTTCGTTGGCACCCCAA	0,02	0,10	0,22
GGTTTCGCCAATCACTACAACGTACGTGCCCGTATG	0,14	0,07	0,17
CCGGTGAGCCTTTCCGCACGCTTGAGCAAGTCACGC	0,18	0,17	0,09
TGTTGTCTGGATTTGCCGATAGTCACGTTACGTAAC	0,12	0,05	0,19
ACAATGTCACTTAAATCCCGGCCCGAATCACGTAAC	0,26	0,16	0,05
CGTATGTCTGACCCTGACATAAAACGTTAGCGTAAC	0,26	0,15	0,05
TGGTTAAGAGCAACTGCCCTAATCTCACAATGCCCC	0,01	0,04	0,22
GGTTGCCTGCATTTACGCTGTTTACGCCTTCGCAGC	0,07	0,07	0,17
CGTGCCGACCCGCATGTCCACGGTGCTCCACACACC	0,21	0,13	0,06
GGTCTGCCTTTTCATATAACCGTTTATCTCACTCCC	0,01	0,08	0,17
GATTGTCCCAAATCATCCTTAGAACTTTTACCTCCA	0,08	0,12	0,12
TACTTACGTTTATGTCCTGCGGTCTACGCCCTCCCC	0,21	0,14	0,04
GTTTGGCTTCCATCCGCGTATCAACCGTCGACCCGT	0,01	0,06	0,17
CGCGCTCGTTAGCAATTTCGGTGTCCCGTTATAATC	0,02	0,09	0,14
GGCGAGCCGCAGCACCGACTTCACGCATACGCGTTC	0,36	0,07	0,02
CTCGGAAAACCGCTGATTTCGCTCACTGACCCCACA	0,07	0,11	0,09
GTTACAAAATGTCCCCGCATGCGTCCACGCTCTCGC	0,14	0,06	0,09
GAGTTGCCCTTATCCTCCCAACGCATGCACCAGCGC	0,13	0,07	0,09
GGAATGCCCGCCTCTAACGCTCAGGCCGTCTGCTGC	0,01	0,06	0,14
CACCCCAACGCCCCTTGCGGACAAGCGTTGTCCTGC	0,05	0,14	0,07
TGTTCCCTTATTGTAAACACACGTGCCCTCGTGTGC	0,10	0,03	0,12
CGCCCATATCACTGGGACTTGACGTATCTTGGCCGC	0,05	0,07	0,11
CGGCTGCCAACCGCATATCGTGAAGGCTGGCTTCGA	0,11	0,08	0,07

GATTGTCCCAAATTATCCTTAGAACCTTTACCTCCA	0,04	0,08	0,10
TCTTGCCGAGTGCGCATCGACAAGTCAGAATGCCCC	0,01	0,03	0,14
GATTGTCCCAAATTATCCTTAGAACTTTTACCTCAA	0,03	0,08	0,10
CGTACAGACGTGCACGCCAAACGTCGTACGGCCTCG	0,01	0,04	0,12
CGCACGCAACGCCGCTGTTCCGTGAGATTGAGCCCT	0,01	0,07	0,10
