# Supplementary material for: Targeting ocular tissues with intravenously administered aptamers selected by in vivo SELEX
Source: Mol Ther Nucleic Acids. 2024 Oct 4;35(4):102352. doi: 10.1016/j.omtn.2024.102352 (PMC11513532; doi:10.1016/j.omtn.2024.102352)
Supplement: Document S2. Article plus supplemental information [file mmc2.pdf]

Molecular Therapy
Nucleic Acids
Original Article

Targeting ocular tissues with intravenously administered aptamers selected by *in vivo* SELEX

Sonja Korhonen,[1] Katja Stenberg,[1] Umair Seemab,[1] Piia Bartos,[1] Katariina Mäkiniemi,[1] Jørgen Kjems,[2] Daniel Miotto Dupont,[2] and Astrid Subrizi[1]

[1]School of Pharmacy, Faculty of Health Sciences, University of Eastern Finland, Yliopistonrinne 1 C, 70210 Kuopio, Finland; [2]Interdisciplinary Nanoscience Center (iNANO), Department of Molecular Biology and Genetics, Aarhus University, Gustav Wieds Vej 14, 8000 Aarhus C, Denmark

Ocular diseases create a significant economic burden and decrease in quality of life worldwide. Drugs and carrier molecules that penetrate ocular tissues after intravenous administration are needed for more efficient and patient-friendly treatment of ocular diseases. Here, ocular barrier-penetrating aptamers were selected through the utilization of *in vivo* SELEX and intravenous injection in rats. Three aptamers—Apt1, Apt2, and Apt5—were chosen based on their specific accumulation in vascularized ocular tissues and further characterized for their *in vivo* biodistribution using quantitative reverse-transcription PCR (RT-qPCR). A statistically significant difference between ΔCt values of ocular and control tissues with Apt2 ($p < 0.0001$) and Apt5 ($p < 0.0001$) was observed. Interestingly, Apt1 was the most abundant aptamer in the sequencing pool, but it did not show a statistically significant difference in *in vivo* biodistribution between ocular and control tissues. Overall, this study established a functional *in vivo* SELEX method for discovering ocular tissue targeting aptamers.

INTRODUCTION

Visual impairment and blindness are increasing health problems, which are predicted to affect over 280 million people globally by 2040 and cause a significant economic burden of billions of euros for the healthcare system.[1] In addition to financial costs, ocular diseases remarkably decrease quality of life.[2] Ocular diseases that cause vision impairment and blindness affect almost all tissues in both the anterior and posterior segments of the eye. Unfortunately, the treatment of ocular diseases is focused on local drug administration either on the surface of or directly into the eye. For instance, repeated intravitreal injections of vascular endothelial growth factor (VEGF) antibodies for the treatment of posterior eye neovascular diseases pose a risk of serious side effects and poor patient compliance.[3] To improve patient compliance and reduce costs and side effects, the route of administration should be appropriate, and the dosing interval should be increased from 4 to 12 weeks to biannually or even less frequently. However, systemic administration of ocular pharmacotherapy requires large amounts of active drug, which increases the probability of serious adverse effects. Furthermore, due to the challenging biopharmaceutical properties and rapid elimination of drugs from the eye, maintaining therapeutic levels inside the ocular tissues is difficult.[4,5] Thus, drugs and carrier molecules that could penetrate ocular tissues after systemic (i.e., intravenous) administration would be important for more efficient and patient-friendly treatment for ocular diseases.

Aptamers are a promising possibility for the active drug targeting of ocular tissues. Aptamers are short, single-stranded RNA- or DNA-based molecules with good stability, low-cost production, and excellent targeting properties.[6] Aptamers can be highly specific and possess excellent affinity toward their target molecules. Aptamers usually interact with their target molecules through multiple interaction points and can form unique three-dimensional structures that cover a large surface area.[7] In addition, aptamers can have a dissociation constant in a low picomolar range.[8] Aptamer therapeutics have already been utilized in the treatment of ocular diseases. Pegaptanib (Macugen), a PEGylated 28-base RNA aptamer inhibiting $VEGF_{165}$, was accepted for the treatment of wet age-related macular degeneration (AMD) in 2004 by the US Food and Drug Administration (FDA).[9] Despite falling behind due to the introduction of more efficient anti-VEGF antibodies, the development of ocular disease targeting aptamers continued, and in 2023, avacincaptad pegol (Izervay), a complement C5 inhibitor, was approved by the FDA for the treatment of geographic atrophy secondary to AMD.[10]

The systematic evolution of ligands by exponential enrichment (SELEX) method established in the 1990s is used for aptamer selection.[11,12] Several rounds of SELEX will eventually yield aptamer sequences with high affinity toward the target. There are multiple SELEX methods available, most notably, protein SELEX and cell SELEX. However, aptamers discovered with these methods may not possess optimal pharmacokinetic or biopharmaceutical properties.[13] In contrast, *in vivo* SELEX performed under complex physiological conditions of living animals is more likely to yield aptamers functional *in vivo*. Thus, the aptamers obtained by *in vivo* SELEX could more likely function in humans. It has been previously demonstrated

Received 17 June 2024; accepted 30 September 2024;
https://doi.org/10.1016/j.omtn.2024.102352.

Correspondence: Sonja Korhonen, School of Pharmacy, Faculty of Health Sciences, University of Eastern Finland, Yliopistonrinne 1 C, 70210 Kuopio, Finland.
E-mail: sonja.korhonen@uef.fi

© 2024 The Author(s).
Published by Elsevier Inc. on behalf of The American Society of Gene and Cell Therapy.
This is an open access article under the CC BY license (http://creativecommons.org/licenses/by/4.0/).

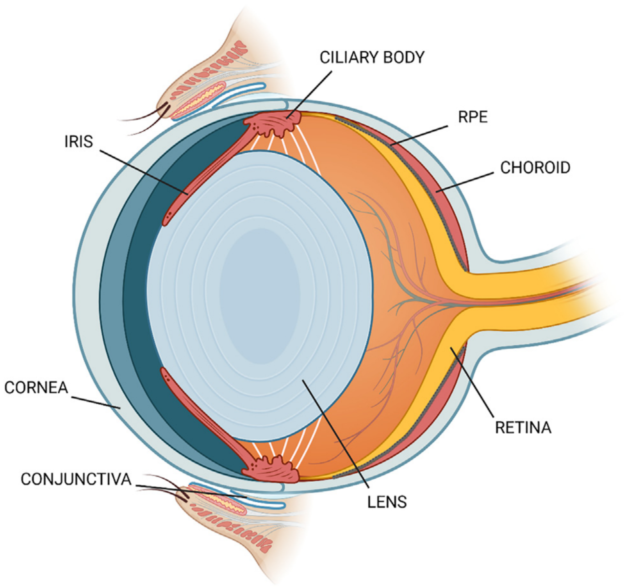

Figure 1. A representative image of rat eye and ocular tissues subjected to *in vivo* SELEX and aptamer discovery

Table 1. Characteristics of the obtained aptamer pool from ocular and control samples pooled from cycles 10, 12, and 14

Filter	No. of sequences
Unique sequences (≥1 copy)	14,482
Unique 36-nt sequences (≥1 copy)	13,164
≥10 counts	11,273
≥100 counts	6,234
≥1,000 counts	329
≥2,000 counts	128
≥5,000 counts	41
≥10,000 counts	16
≥20,000 counts	12
≥30,000 counts	9
≥40,000 counts	6
≥100,000 counts	4
≥200,000 counts	3
≥1 million counts	1

The number of unique sequences with the correct 36-nt variable region depends on the applied copy-number filter.

that *in vivo* selected brain-targeting aptamers could cross the tight blood-brain barrier (BBB).[14] As the BBB and the blood-retina barrier resemble one another,[15] *in vivo* SELEX could be viable for discovering ocular-targeting aptamers. Such *in vivo* selection of ocular-targeting aptamers using an intravenous administration route has never been conducted before.

This study presents a selection of ocular barrier penetrating aptamers from different ocular tissues using *in vivo* SELEX and intravenous injection.

RESULTS

Characterization of the NGS aptamer pool from SELEX cycles 10, 12, and 14

A selection of ocular barrier-penetrating aptamers was conducted using a 2′-fluoropyrimidine (2′F)-modified RNA pool and utilizing *in vivo* SELEX and intravenous injection in rats. A total of 14 *in vivo* SELEX cycles were performed by extracting aptamers from ocular and control tissues. Ocular tissues investigated in this *in vivo* SELEX were conjunctiva, cornea, iris-ciliary body (cb), lens, retina, and retinal pigment epithelium (RPE)-choroid (Figure 1). Every second SELEX cycle was subjected to next-generation sequencing (NGS) to discover ocular tissue-targeting aptamers. Based on previous *in vivo* SELEX publications, sequenced *in vivo* SELEX cycles 10, 12, and 14 were subjected to more thorough subsequent analysis.[14,16,17] The characterization of the initial sequencing pool consisting of these three cycles yielded a total of 14,482 unique sequences with ≥1 counts. The characteristics of the SELEX pool are presented in Table 1. From these, 13,164 sequences possessed 36-nt-long variable sequences. The total number of correct 36-nt sequence counts obtained in cycles 10, 12, and 14 were 1,132,702, 1,746,830, and 2,771,101, respectively. The number of unique sequences decreased when the minimum abundance threshold was increased gradually from ≥1 to ≥1 million counts. For instance, the number of unique sequences decreased by ∼34-fold when the minimum abundance threshold was increased from ≥10 to ≥1,000 counts (11,273 vs. 329 sequences, respectively). The minimum abundance threshold of ≥100,000 counts resulted in only 4 unique sequences. In addition, only 1 sequence exceeded ≥1 million sequence counts.

A family search of the aptamer pool from SELEX cycles 10, 12, and 14 was performed. Aptamer families can be defined in multiple ways, but our family search focused on sequences with >80% nucleotide similarity. This search resulted in 5 different aptamer families aligned using LocARNA to visualize the stem-loop structure. The number of members varied between the discovered aptamer families: family A consisted of 90 members and 37.3% of total sequence counts in SELEX cycles 10, 12, and 14. These values for other families were the following: family B: 21 members and 4.0%; family C: 32 members and 4.4%; family D: 11 members and 2.37%; and family E: 5 members and 0.58%. Interestingly, the most enriched aptamers obtained from these families were among the 8 most enriched sequences overall. The most abundant sequence (variable region GATTGTCCCAAATTAT CCTTAGAACTTTTACCTCCA, Apt1, family A) made up 34.53% of all sequence counts. The next 7 most abundant sequences made up 3.89% (variable region TTGACTGAATACGCACATTCG CCAAATTGCCGGCCC, Apt2, family B), 3.48% (variable region ATGCCGTCCGACCCACTCGTACGGCACTATCTCCCC, Apt3, family C), 2.33% (variable region CAATGCCCTCATGTTTTTGC TCAAAACATCACTGCT, Apt4, family D), 0.73% (variable region TGTACGCTCGCATTTGTGCGTTGGTGACCGCACTCA, Apt5),

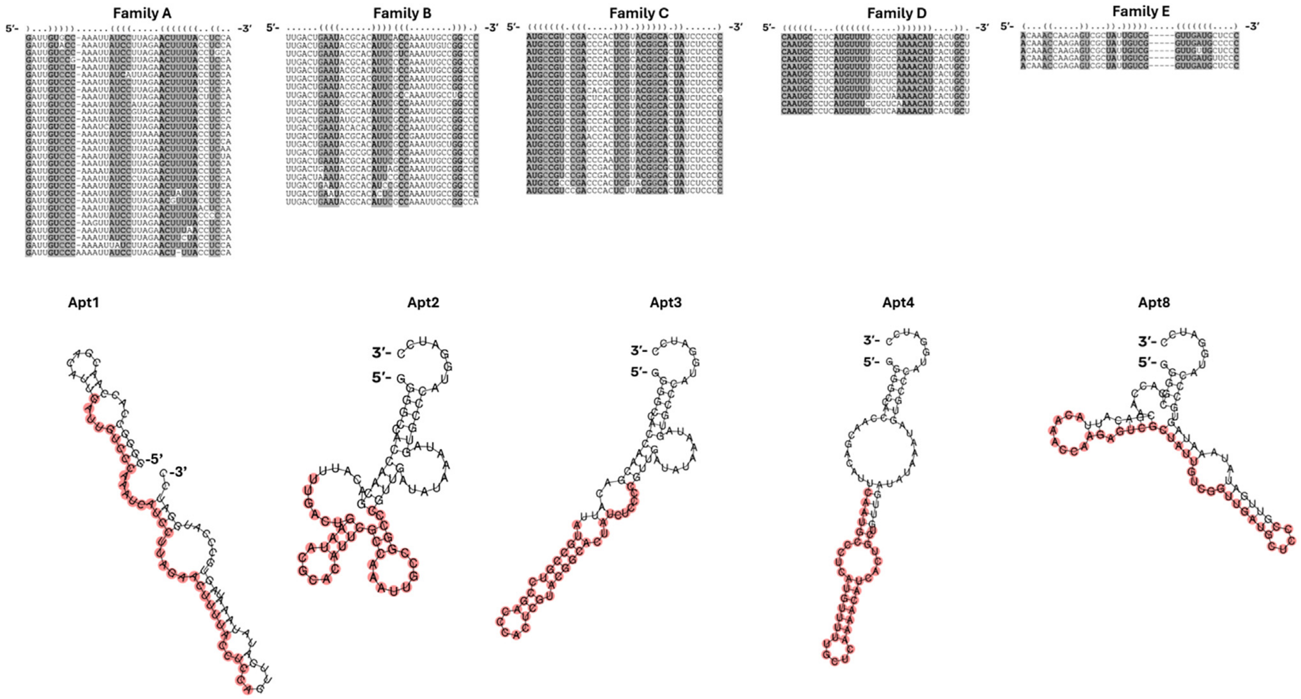

Figure 2. Families with >80% nucleotide conservation were discovered from the *in vivo* SELEX dataset consisting of SELEX cycles 10, 12, and 14

LocARNA was used for the alignment of sequences and identification of stem and loop structures. Because of the inner limit of LocARNA, only the 30 most enriched sequences were aligned. The consensus sequence is shown with dot-bracket notation, where dots represent loops and brackets stem regions. Conserved bases within the family in the stem region are highlighted in gray. For clarity, constant regions are omitted from the alignment. Family A consisted of 90 members, B of 21 members, C of 32 members, D of 11 members, and E of 5 members. The most enriched aptamer from each family was folded with RNAfold WebServer. The name, MFE predicted structure, and variable region highlighted in red are shown under the respective LocARNA alignment.

0.67% (variable region GATTGTCCCAAATTATCCTTAGAACTTTTATCTCCA, Apt6, family A), 0.54% (variable region TCTGCTAGGCGGGATTTTCGTCAGTGTCCCACCCAT, Apt7), and 0.54% (variable region ACAAACCAAGAGTCGCTATTGTCGGTTGATGCTCCC, Apt8, family E) of all sequence counts pooled from cycles 10, 12, and 14 (Table S1). The abundances of individual aptamer sequences varied between different SELEX cycles (Table S2). Apt1 and Apt6 were enriched in family A, Apt2 was the most enriched member of family B, Apt3 was the most enriched member of family C, Apt4 was the most enriched member of family D, and Apt8 was the most enriched member of family E. The minimum free energy (MFE) structure predicted with RNAfold WebServer of the most enriched aptamer from each family is shown next to the respective LocARNA aptamer family alignment (Figure 2). The predicted MFE structures and LocARNA alignments varied between different enriched aptamer families. Nevertheless, the loop regions were highly conserved within each family.

In addition, the MEME suite was used to identify conserved motifs and potential differences from the 50 most enriched sequences from each tissue (Figure 3). Either 1 (conjunctiva, retina, lens, liver, kidney) or 2 (cornea, iris-cb, RPE-choroid, lung, spleen) 23- to 36-nt-long conserved motifs with E-values lower than 0.05 were discovered from each tissue when the 50 most enriched sequences from each tissue were investigated (Figure 3). The 36-nt-long GATTGTCCCAAATTATCCTTAGAACTTTTACCTCCA motif with a nucleic acid composition of the variable region of Apt1 was extremely conserved and was prominent in highly vascularized control tissues, including the liver, lung, kidney, spleen, and ocular RPE-choroid. When motifs were compared with each other a less conserved Apt1 motif-resembling motif with greater variability in nucleotide composition was also found in the conjunctiva, cornea, and retina (Figure 3). For instance, in position 2 of this motif, there is an additional G alongside the A. The motifs from the iris-cb did not show obvious similarities with the Apt1 motif. In addition, the motif obtained from the lens showed a high degree of variability. These differences are explained by the different distribution of sequence abundance between tissues (Figures 4 and 5).

The abundances of single aptamers vary between SELEX cycles and different tissues

Further investigation was conducted on the 8 most enriched sequences regarding the composition of the sequence pool and the variations in sequence abundances between SELEX cycles (Figure 4). Overall, there were substantial differences in the sequence abundances between these 8 aptamer sequences. Apt1, the most enriched sequence, was more abundant in ocular tissues in cycles 10 and 12 (19.52% and 27.88%, respectively) than in control tissues (6.58%

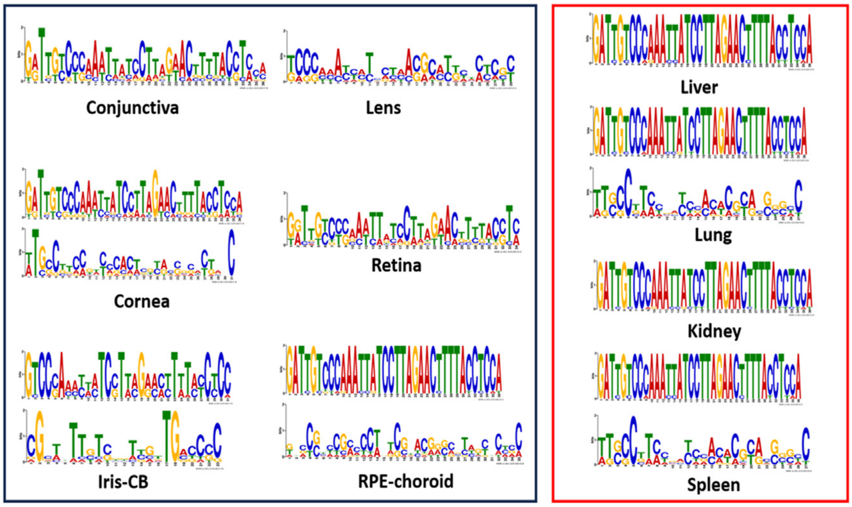

Figure 3. Significant consensus motifs from the 50 most enriched sequences per tissue

Ocular tissues are outlined with black and control tissues with red. From conjunctiva, retina, lens, liver, and kidney, 1 significant consensus motif with an E-value under 0.05 was discovered. An E-value of 0.05 depicts a 5% chance for an alignment to occur randomly. From cornea, iris-cb, RPE-choroid, lung, and spleen, 2 conserved motifs with an E-value under 0.05 were discovered.

and 12.16%, respectively). However, in the last sequenced cycle, Apt1 was more abundant in control tissues (25.29%) than in ocular tissues (19.09%). Apt6, a sequence closely related to Apt1, displayed a similar pattern to Apt1, but the abundance in the sequence pool remained under 0.6% in both ocular and control tissues. The abundance of Apt2 in the sequence pool consistently increased from cycle 10 to the last sequenced cycle. Apt2 was more abundant in ocular tissues (3.17%) than in control tissues (2.50%) in the last sequenced cycle. Apt 8 displayed a similar pattern to Apt2, but its abundance was remarkably lower, remaining below 0.5% of the pool in cycles 10, 12, and 14. The sequence abundance of Apt3 was higher in ocular tissues than in control tissues. However, its sequence abundance dropped from cycle 12 to the last sequenced cycle in both ocular (2.87%–1.58%) and control tissues (2.24%–1.19%), respectively. The same trend was also observed in the sequence abundance of Apt4. Interestingly, the sequence abundance of Apt5 increased significantly in the last sequenced cycle, especially in ocular tissues (0.02%, 0.09%, and 1.21% in cycles 10, 12, and the last cycle pool, respectively), but also in control tissues (0.005%, 0.017%, and 0.31% in cycles 10, 12, and the last cycle pool, respectively). This same phenomenon was seen with Apt7, but its total abundance was lower than with Apt5.

Sequence abundances in the pools varied significantly between different aptamers and SELEX cycles. Thus, the tissue distribution of these aptamer sequences was investigated further using the SELEX cycle sequenced last, as the aptamer abundance changed significantly from cycle 10 to the last sequenced cycle (Figure 4; Table S2). The aptamer sequence counts from the last sequenced cycle per tissue type were normalized to the total counts obtained from each tissue. Overall, the lowest sequence abundance was observed in the lens, which is an avascular tissue (Figure 5). This pattern was observed with all investigated aptamers (Apt1–Apt8). Apt1, the most abundant aptamer, presented the highest enrichment in the kidney, RPE-choroid, and iris-cb (59.44%, 53.82%, and 43.58% of total sequence counts per tissue, respectively). Overall, Apt1 dominated the selection, comprising 34.53% of the sequence pool (Tables S1 and S2), and having the highest abundance in each tissue (Figure 5). Apt2 showed the highest enrichment in the retina (12.11% of total retina sequence counts), which differed from other investigated aptamers. Apt3 and Apt6 were distributed between ocular and control tissues, with the highest percentage in iris-cb encompassing a total of 4.55% and 1.73% of iris-cb sequence counts, respectively. Apt4 encompassed 3.02% of RPE-choroid sequence counts. However, the results regarding the sequence distribution of the RPE-choroid are from SELEX cycle 12, and therefore, any potential change to cycle 14 is impossible to infer. Interestingly, Apt5 and Apt7 showed the highest abundance in the iris-cb (6.19% and 3.69% of counts, respectively), which was notably different when compared to other tissues. Apt8 had the highest percentage in iris-cb (1.26% of counts).

In vivo biodistribution study reveals different ocular-targeting properties of individually administered aptamers

As the NGS data allow for relative comparison of sequence pools between different cycles and tissues, the performance and targeting of individual aptamers must be verified with quantitative methods. Thus, we decided to perform a proof-of-concept *in vivo* biodistribution study, where aptamers were administered individually to compare their targeting properties. Apt1, Apt2, and Apt5 were chosen for this study and quantitative reverse-transcription PCR (RT-qPCR) was used for the quantification of aptamers from each tissue. The selection for the *in vivo* biodistribution study was based on different tissue-targeting patterns obtained from the NGS data. Apt1 was the most dominating sequence, Apt2 was the only sequence from the 8 most abundant sequences to encompass the highest percentage in the retina, and the distribution of Apt5 indicated preferential homing to iris-cb (Figure 5). The amount of cDNA was normalized to 4 ng and was identical in all samples subjected to RT-qPCR. The normalization procedure was similar to the *in vivo* SELEX and NGS sample preparation. The obtained cycle threshold (Ct) values from Apt1, Apt2, and Apt5 were normalized to Ct values of a control aptamer (Control apt) from respective tissues and presented as ΔCt, indicating the change in abundance compared to Control apt ($\Delta Ct = Ct_{Aptamer} - Ct_{Control\ apt}$) (Figures 6 and 7). As the PCR reaction is exponential, a 4-unit difference in Ct values would translate to a 16-fold difference in aptamer abundance.

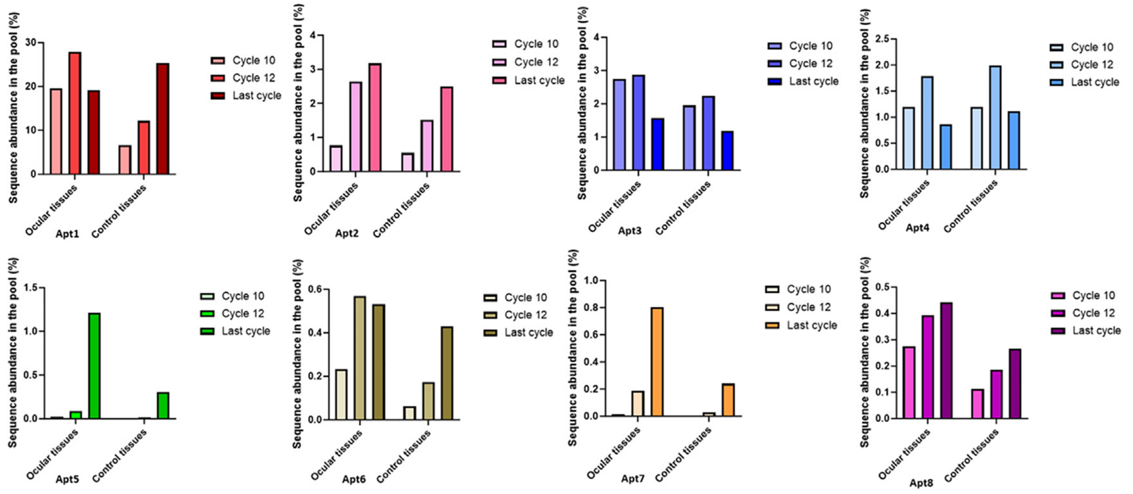

Figure 4. The variation of sequence abundance of the 8 most enriched aptamers in ocular and control tissues during the 10, 12, and last sequenced SELEX cycle

The sequence counts were normalized to the total counts obtained from SELEX cycles 10, 12, and 14. The last cycle contains cycle 12 for RPE-choroid and cycle 14 from other tissue samples. Note the difference in the axis scales between each aptamer.

The ΔCt values of Apt1, Apt2, and Apt5 were calculated for ocular tissues (conjunctiva, cornea, iris-cb, lens, retina, and RPE-choroid) and control tissues (liver, lung, kidney, and spleen) (Figure 6). Overall, Apt2 possessed higher ΔCt values with statistically significant differences when compared to Apt1 and Apt5 in samples obtained from the conjunctiva, cornea, lens, and retina ($p < 0.0001$). In addition, Apt5 possessed statistically significant differences and higher ΔCt values than Apt1 and Apt2 in iris-cb, RPE-choroid, and lung tissues ($p < 0.0001$). Apt1 did not show any statistically significant ΔCt values higher than those of Apt2 or Apt5.

RT-qPCR data from ocular tissues (conjunctiva, cornea, iris-cb, lens, retina, and RPE-choroid) and control tissues (liver, lung, kidney, and spleen) were further pooled together to highlight potential differences between the ocular tissues and well-perfused organs (Figure 7). The ΔCt values for Apt1 in ocular tissues were 0.70 and in control tissues 0.55, and there were no statistically significant differences between ocular and control tissues ($p = 0.7897$). There was a statistically significant difference between ocular and control tissues in Apt2 ΔCt values (3.43 and 0.07, respectively, $p < 0.0001$) and Apt5 ΔCt values (2.47 for ocular tissues and −0.01 for control tissues, $p < 0.0001$). Furthermore, there was a statistically significant difference between the ocular ΔCt values of Apt1 and Apt2 ($p < 0.0001$) and Apt1 and Apt5 ($p = 0.0013$). There was no statistically significant difference between the ocular ΔCt values of Apt2 and Apt5 ($p = 0.1478$).

The 3-dimensional (3D) structures of the preferentially ocular tissue-targeting aptamers Apt2 and Apt5 were built based on *de novo* folding guided by deep learning restraints with the trRosettaRNA web server. The confidence levels for the models were either medium or low, indicating uncertainty in the predicted folds. Both aptamers were predicted to have a looping double-helical structure at their 5′ terminal, whereas the 3′ terminal was predicted to be less structured (Figure S1). The circular dichroism (CD) spectra of aptamers Apt2 and Apt5 resembled these predicted structures. The CD spectrum of Apt2 exhibited peaks resembling a possible double-helical structure, with a maximum at 265 nm and a minimum at 210 nm. In contrast, the spectrum of Apt5 displayed a less pronounced peak at 210 nm, indicating uncertainty in its folded form. The melting temperatures of Apt2 and Apt5 were measured and observed to be 58.92°C and 63.40°C, respectively (Figure S2).

DISCUSSION

The systemic administration of drugs is used in the management of some ocular diseases. For instance, small-molecule drugs such as carbonic anhydrase inhibitors are used clinically for the treatment of elevated intraocular pressure, and antibiotics are used against ocular infections.[18,19] Even macromolecules are administered systemically in cases of serious ocular diseases such as uveitis.[20] Nevertheless, there are severe systemic side effects related to these treatment options originating from the non-targeted nature of these therapies. Thus, a majority of ocular diseases are treated using local administration, especially intravitreal injections, when the retina is targeted.[5] These intravitreally administered macromolecular therapeutics include RNA aptamer-based avacincaptad pegol and pegaptanib.

The penetration of macromolecules into ocular tissues is limited due to the blood-retina and blood-aqueous barriers that protect the ocular tissues from exogenous compounds.[5] The fenestrated capillaries in the stroma of ciliary processes enable limited penetration of macromolecules to the anterior segment of the eye.[21] As a result, the overall insufficient exposure limits efficient usage of intravenous therapeutics for posterior ocular tissues such as the retina and anterior tissues with poor penetration. Hence, the challenge with systemic ocular

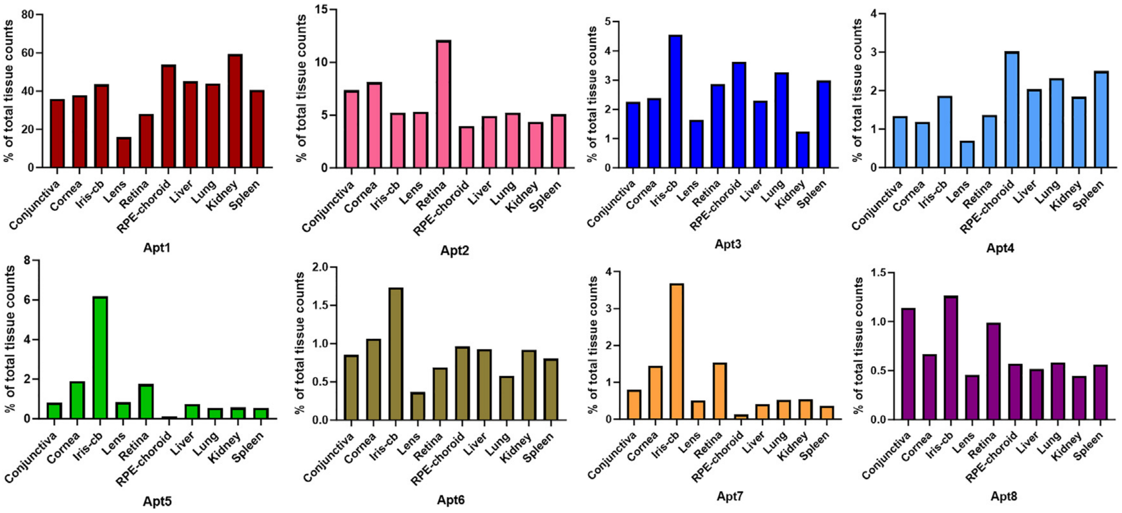

Figure 5. Percentages of the 8 most enriched aptamers in each tissue from the last sequenced SELEX cycle
The aptamer sequence counts from each tissue were normalized to the total counts obtained from the respective tissue. The RNA obtained from each tissue was normalized to equimolar amounts. The last cycle counts contain cycle 12 for RPE-choroid and cycle 14 from other tissue samples. Note the difference in the axis scales between each aptamer.

pharmacotherapy is to reach therapeutically meaningful concentrations and residence time in the target compartment, which is especially difficult to achieve with macromolecular drugs. The goal in our study was to discover ocular barrier-penetrating aptamers utilizing the *in vivo* SELEX method.

In vivo SELEX is a relatively new aptamer selection technique compared to *in vitro* SELEX methods. Despite a multitude of aptamers having been selected using *in vitro* SELEX techniques, *in vivo* functionality is not guaranteed with *in vitro* selected aptamers.[22] Sequences selected using *in vivo* SELEX are "forced" to have favorable pharmacokinetic properties that permit target homing, whereas such properties may be lacking in *in vitro* selected aptamers. This is important especially if the target is in a pharmacokinetically challenging organ such as in the CNS or in the eye. Usually, *in vitro* SELEX protocols require an additional negative selection step excluding aptamers that are binding, for example, to the immobilization matrix.[13] In addition, the stringency of selection is crucial for the discovery of high-affinity aptamers and is usually increased by varying the target concentration.[23] However, these features are inherently built in *in vivo* SELEX. Furthermore, the exact targets of the aptamers do not need to be known when utilizing *in vivo* SELEX, which in addition to targeting aptamer discovery could enable the discovery of new target molecules.

In our *in vivo* SELEX method, we utilized a 2′F-modified RNA aptamer library, which comprised >10^{15} RNA sequences. The 2′F-modification in the RNA molecule is known to increase the binding affinity and nuclease resistance of these molecules.[8,13] Furthermore, RNA aptamers can adopt more variable conformations than DNA aptamers, further diversifying the used aptamer pool.[24] The reduced degradation of RNA combined with higher binding affinity and structural diversity also increased the possibility of discovering ocular targeting aptamers from our pool. In addition, traditional Sanger sequencing is far less sensitive compared to modern NGS methods.[25] It is also known that aptamers with less structural stability outcompete aptamers with high structural stability in PCR, which could skew the SELEX process.[26] The NGS used in our study enabled a detailed analysis of recovered aptamer sequences from each tissue sample. Thus, the risk of overlooking less-abundant sequences due to skewing was decreased. Despite the fact that Apt1 dominated the sequence pool, a multitude of other sequences with the correct 36-nt-long variable region were also recognized.

Even though the *in vivo* SELEX NGS pool was dominated by Apt1, further investigation regarding the tissue distributions and variations in sequence abundances between SELEX cycles revealed interesting differences. The sequence abundance of Apt2 (3.89% of sequence pool) was constantly higher in ocular tissues than in control tissues in all sequenced cycles, whereas Apt1 was most abundant in the control tissues in the last sequenced cycle (Figure 4). Interestingly, the abundance of Apt5 (0.73% of sequence pool) increased by ~60-fold from cycle 10 to cycle 14. This increase could have been even greater if the RPE-choroid sample had been included from cycle 14. In addition, differences in tissue distributions among the 8 most enriched aptamers were observed (Figure 5). The lowest number of sequences was obtained from the lens, which was to be expected because of the lack of vascularization. The dominant sequence, Apt1, was most abundant in the kidney, while Apt2 was most abundant in the retina. Although the abundance of Apt5 in the sequence pool was not high (0.73%), it was responsible for 6.19% of iris-cb sequence counts in cycle 14. Nevertheless, despite the clear enrichment of ocular targeting

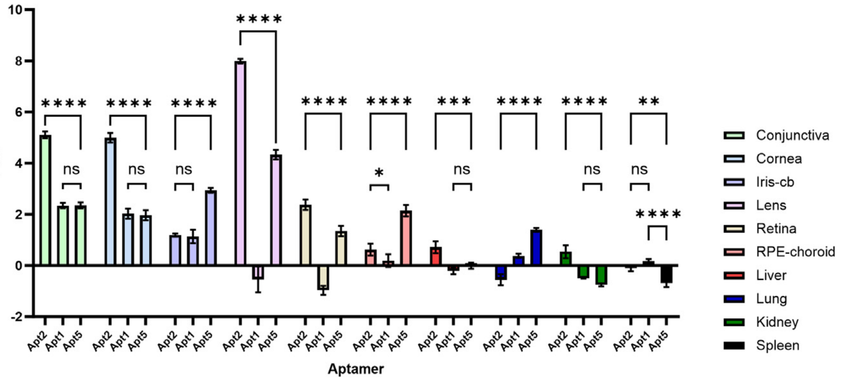

Figure 6. The calculated Ct values from Apt1, Apt2, and Apt5 from the conjunctiva, cornea, iris-cb, lens, retina, RPE-choroid, liver, lung, kidney, and spleen normalized to Ct values of a control aptamer and presented as ΔCt ($\Delta Ct = Ct_{Aptamer} - Ct_{Control\ apt}$)

Higher ΔCt values indicate higher aptamer abundance compared to the control aptamer and negative values indicate lower abundance compared to the control aptamer. The PCR is exponential and, for example, an increase of 4 units in the Ct value depicts a 16-fold difference in the original PCR template. Data were analyzed using a 2-way ANOVA with Tukey's multiple comparisons test and are presented with mean and SD. *$p < 0.05$; **$p < 0.01$; ****$p < 0.0001$; ns, not significant.

sequences observed in the NGS data, only changes in the sequence pool between different cycles and tissues can be compared. Determining the actual quantities of sequences in the target tissues without further experiments is challenging, since NGS only produces information about the preferential homing of different sequences. Furthermore, it is important to verify that when a pool consisting of multiple different sequences is reduced to the administration of a single aptamer, the targeting properties and performance of promising aptamers remain the same. Thus, the performance and targeting of three different profile aptamers—Apt1, Apt2, and Apt5—administered individually were verified using RT-qPCR.

The results of RT-qPCR were normalized to a Control apt, which was a sequence within the original aptamer pool that was undetectable by NGS in the last sequenced SELEX cycles. Since Control apt is a non-ocular selective sequence, its targeting of ocular tissues should be reduced, but its levels in control tissues should be in the same range as or higher than those of the ocular targeting aptamers Apt1, Apt2, and Apt5. Overall, the ΔCt values of Apt2 and Apt5 were positive and higher in ocular tissues than in control tissues (Figure 6). Apt2, which showed higher abundance in the retina in the NGS pool, also had the highest ΔCt value in the retina of investigated aptamers in the *in vivo* biodistribution study. The *in vivo* SELEX NGS results regarding the abundance of Apt5 correlated with ΔCt, since Apt5 had the highest ΔCt value of investigated aptamers in iris-cb. Moreover, Apt5 was pronounced in RPE-choroid, a highly vascularized, pigmented tissue similar to iris-cb. Interestingly, Apt1, a sequence that dominated the NGS sequence pool, did not perform well in the *in vivo* biodistribution experiment and had the lowest ΔCt values of all investigated aptamers in ocular tissues. The pooled ΔCt values from ocular tissues further highlighted the significant differences among these three aptamers. The ΔCt values of Apt2 and Apt5 from ocular tissues were significantly different from those of control tissues. No such statistical difference could be detected with Apt1 (Figure 7). The high abundance of Apt1 in the NGS pool is most likely due to overpowering PCR performance or a dependence on pooled administration, leading to poor performance when administered individually. This highlights the importance of the deep and thorough NGS method for the recognition of less-abundant aptamers in the SELEX pool. This also demonstrates that the most abundant aptamer does not necessarily perform well, and that less-dominating aptamers are important to consider for further downstream studies. Thus, despite lower abundance in the NGS pool, Apt2 and Apt5 showed statistically significant homing to ocular tissues. These aptamers overcome the poor macromolecule penetrance to ocular tissues after intravenous injection.

The *in vivo* biodistribution study proved that the selected aptamers Apt2 and Apt5 were targeting different ocular tissues after intravenous injection and a 30-min circulation time. The pharmacokinetic properties of these aptamers could be further enhanced with different modifications to enable longer circulation time and reduced clearance. PEGylation can increase the half-life of aptamers from minutes to hours when administered intravenously (reviewed extensively by Kovacevic et al.[8]), but it led to severe allergic reactions due to preexisting anti-polyethylene glycol (PEG) antibodies, resulting in the termination of the clinical trial.[27,28] Alternatives for PEGylation for the improvement of aptamer pharmacokinetics are therefore needed. One possible alternative is aptamer-cholesterol conjugation, which in a hepatitis C virus-targeting aptamer resulted in a 2-fold increase in half-life and a 9-fold reduction in clearance.[29] Nevertheless, the modification should always be chosen with the target tissue in mind. With ocular-targeting aptamers, the correct conjugation strategy that, for instance, could enhance the penetration through the blood-retina barrier, could increase their therapeutic potential. In the future, a more detailed pharmacokinetic analysis of the behavior of these aptamers could be performed since saturable and/or nonlinear pharmacokinetics of variable aptamers have been reported previously.[30,31] A thorough characterization of the aptamer target molecules could help with the identification of the ocular-targeting pathways and pharmacokinetic factors after intravenous injection.

Here, we demonstrated an *in vivo* SELEX process targeting ocular tissues in rats and further verified *in vivo* targeting properties of individually administered aptamers Apt2 and Apt5. This study shows the feasibility of targeting complex and difficult-to-reach ocular tissues with large and negatively charged macromolecules. The aptamer pool generated here could be used to compare SELEX pools from various ocular disease models and different species. In the future, well-characterized ocular barrier-penetrating aptamers could be

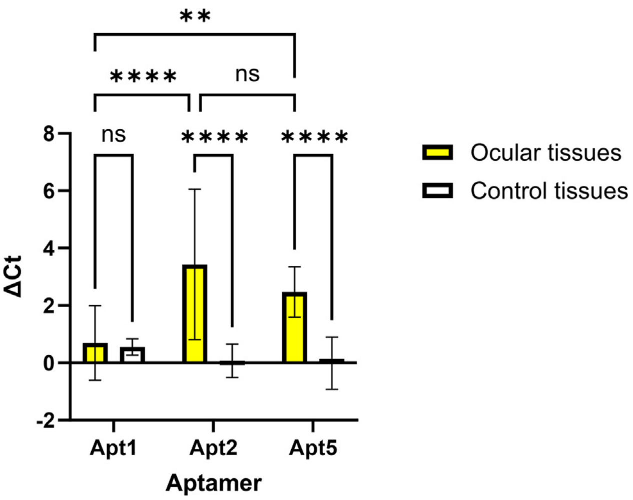

Figure 7. Pooled ΔCt values from the eye (conjunctiva, cornea, iris-cb, lens, retina, RPE-choroid) and well-perfused organs (liver, lung, kidney, spleen) from Apt1, Apt2, and Apt5

Ct values of each aptamer are normalized to Ct values of a control aptamer and presented as ΔCt ($\Delta Ct = Ct_{Aptamer} - Ct_{Control\ apt}$). Higher ΔCt values indicate higher aptamer abundance compared to the control aptamer, and negative values indicate lower abundance compared to the control aptamer. The PCR is exponential, and an increase of 4 units in the Ct value depicts, for example, a 16-fold difference in the original PCR template. Data were analyzed using a 2-way ANOVA with Tukey's multiple comparisons test and are presented with mean and SD. $^{**}p < 0.01$; $^{****}p < 0.0001$; ns, not significant.

utilized as drug carriers enabling more efficient use of currently available therapeutics and diverse aptamer-cargo combinations for the treatment of ocular diseases.

In conclusion, we present for the first time a selection of ocular-barrier penetrating aptamers from various ocular tissues using *in vivo* SELEX and intravenous injection in rats. The targeting properties of selected individual aptamers were further characterized in a proof-of-concept *in vivo* biodistribution study. Overall, this study established an ocular-targeting functional *in vivo* SELEX method, which can be further applied to other aptamer libraries, disease models, and in the delivery of therapeutics to the eye.

MATERIALS AND METHODS

The initial RNA aptamer library

The serum-stable 2′F –modified RNA pool was prepared as described previously by Fjelstrup et al. and Madsen et al.[32,33] A pool of aptamers with the structure 5′-GGGGCCACCAACGACAUU-N36-GUUGAU AUAAAUAGUGCCCAUGGAUC-3′ (N36 denoting a 36-nt-long variable region flanked by constant regions) was produced. Overall, the final aptamer pool consisted of $>10^{15}$ RNA sequences.

Animals

Adult male (4–12 weeks old) HsdOla:LH pigmented rats (Envigo Laboratories B.V., Indianapolis, IN) were used in this study. All animal experiments were performed by the ARVO Statement for the Use of Animals in Ophthalmic and Vision Research and approved by the Finnish National Animal Experiment Board (ESAVI/27769/2020). All rats were housed in 12-h light-dark conditions and were provided with food and water *ad libitum*. All animal experiments were conducted under a deep plane of anesthesia by subcutaneous injection of 0.26 mg/kg medetomidine (Domitor, Orion Oy, Espoo, Finland) and 40 mg/kg ketamine (Ketaminol, Intervet International B.V., Boxmeer, the Netherlands).

In vivo SELEX

The first *in vivo* selection cycle was performed by dissolving the initial RNA library in sterile nuclease-free (nf) water (catalog no. AM9937, Life Technologies, Carlsbad, CA) and heating the library at +85°C for 2 min. The library was then transferred on ice and supplemented to a final concentration of 1× PBS and 5 mM $MgCl_2$. After inducing deep plane anesthesia, 5 nmol of the initial RNA library was injected into the rat tail vein and allowed to circulate for 30 min. For subsequent selection cycles, the injected RNA amount was 0.39 nmol in cycle 2, 1.1 nmol in cycle 3 due to sequence yield, and 1.2 nmol from cycle 4 onward.

After the 30-min circulation period, the rats were euthanized with CO_2 and immediately perfused through the left heart ventricle with 100 mL 1× PBS supplemented with 5 mM $MgCl_2$. The perfusion efficiency was monitored with the bleaching of liver tissue. After perfusion, the eyes were enucleated, and the ocular tissues were dissected under a microscope. From each cycle conjunctiva, cornea, iris-cb, lens, retina, and RPE with choroid were collected from both eyes. Control samples were collected from highly vascularized tissues liver, kidney, lung, and spleen. After collection, all the tissue samples were snap-frozen and stored at −80°C for RNA extraction and amplification. The *in vivo* selection process was repeated 14 times using RNA aptamers recovered and amplified from previous selection cycles.

Tissue homogenization

The collected rat tissue samples were placed in soft tissue homogenizing mix tubes (19–627, OMNI International, Kennesaw, GA) (iris-cb body, retina, RPE-choroid, liver, kidney, spleen) or hard tissue homogenizing mix tubes (19-040E, OMNI International) (conjunctiva, cornea, lens, lung) depending on the tissue type. For lysis, 700 μL QIAzol lysis reagent (catalog no. 1023537, Qiagen, Hilden, Germany) was added to the homogenizing tubes, and the homogenization was performed with Bead Ruptor 24 Elite (catalog no. SKU 19-040E, OMNI International) connected to a Bead Ruptor Cryo cooling unit (catalog no. 19–8010, OMNI International) using programs 2 mL, 6 m/s, 30 s × 3 cycles and 2 mL, 5 m/s, 30 s × 2 cycles for hard and soft tissues, respectively.

Total RNA extraction

The total RNA from each tissue was extracted using the RNeasy Mini Kit (catalog no. 74104, Qiagen) and RNeasy Micro Kit (catalog no. 74004, Qiagen) according to the manufacturer's instructions. Due to the small tissue volume, the RNeasy Micro Kit was used for extracting RNA from the RPE choroid. Briefly, tissue homogenates were

incubated at room temperature for 5 min, and 140 μL chloroform was added and shaken vigorously for 15 s. After a 3-min incubation at room temperature, the homogenates were centrifuged for 15 min at 12,000 × *g* at +4°C. The upper phase was collected, combined with 100% ethanol, and centrifuged at 8,000 × *g* for 15 s at room temperature through RNeasy columns. Genomic DNA was digested with RNase-free DNase Set (catalog no. 79254, Qiagen) at room temperature for 15 min. The total RNA was eluted from the column in nf water and the concentration was measured with a DeNovix DS-11 FX + Spectrophotometer/Fluorometer (DeNovix, Wilmington, DE).

Reverse transcription and PCR amplification

RNA from each sample was reverse transcribed to cDNA. Briefly, forward (5′-CGCGGATCCTAATACGACTCACTATAGGGGCCAC CAACGACATT-3′, desalt purified, Sigma-Aldrich, St. Louis, MO) and reverse (5′-CCCGACACCCGCGGATCCATGGGCACTATTT ATATCAA-3′, desalt purified, Sigma-Aldrich) primers, deoxynucleotide triphosphate mix (10 mM each, catalog no. R0191, Thermo Fisher Scientific, Waltham, MA), and 2 μg RNA per tissue in nf H_2O were heated for 5 min at 65°C, followed by the addition of 200 U SuperScript IV Reverse Transcriptase (catalog no. 18090050, Thermo Fisher Scientific) and incubation at 50°C for 10 min, and followed by inactivation of the reaction at 80°C for 10 min. For double-stranded DNA (dsDNA) production, a Phusion High-Fidelity DNA Polymerase PCR kit (catalog no. F553, Thermo Fisher Scientific) and forward and reverse primers described above were used in the PCR reaction. The optimal number of amplification cycles was determined by running the products in 1.2% UltraPure Agarose gel (catalog no. 16500-100, Invitrogen, Carlsbad, CA) in 1× Tris/borate/EDTA (TBE 10× Liquid Concentrate, catalog no. 0658-1L, VWR, Radnor, PA) for 20 min at 120 V. The gel was imaged with Bio-Rad Gel Doc EZ Imager (Bio-Rad, Hercules, CA). dsDNA was digested with FastDigest BamHI (catalog no. FD0055, Thermo Fisher Scientific), purified using GeneJET PCR Purification kit (catalog no. K0720, Thermo Fisher Scientific), and transcribed to RNA in a reaction containing 80 mM HEPES (pH 7.5), 30 mM DTT, 25 mM $MgCl_2$, 2 mM spermidine-HCl, 2.5 mM ATP and guanosine triphosphate (Thermo Fisher Scientific), 2.5 mM of 2′F-deoxycytidine triphosphate and 2′F-2′-deoxyuridine 5′-triphosphate (MetkinenChemistry, Kuopio, Finland), 100 μg/mL BSA (Thermo Fisher Scientific), 3.5 μM dsDNA template, and 50 μg/mL mutant T7 RNA polymerase Y639F, prepared as described by Krissanaprasit et al.[34]

Purification of RNA with 8% PAGE

The transcribed RNA pool was mixed with formamide gel-loading buffer containing 95% deionized formamide (BioUltra, ≥99.5%, catalog no. 47671, Sigma Aldrich) and 5 mM EDTA (pH 8, 0.5 M in H_2O, catalog no. AM9260G, Thermo Fisher Scientific) and then purified using 8% PAGE in 1× TBE running buffer for 30 min (35 W and 400 mA). The RNA product was cut from the gel under UV light. The RNA was incubated overnight in 0.3 M sodium acetate (3 M, pH 5.5, RNase-free, catalog no. AM9740, Invitrogen), followed by ethanol precipitation, purification, and elution to nf water. The concentrations were measured with a DeNovix DS-11 FX + Spectrophotometer/Fluorometer. For subsequent *in vivo* administration cycles 0.2 nmol of amplified RNA aptamer pool from each ocular tissue (conjunctiva, cornea, iris-cb, lens, retina, and RPE) were pooled together for intravenous injection in the next SELEX round. The total injected aptamer pool amount was 1.2 nmol.

NGS

dsDNA after every second *in vivo* SELEX round was sequenced using NGS. Briefly, the PCR products were elongated and barcoded using Nextera XT Index Kit (24 indexes, 96 samples, catalog no. FC-131-1001, Illumina, San Diego, CA) and KAPA HiFi HotStart ReadyMix (catalog no. KK2601, Roche, Basel, Switzerland) according to the manufacturer's instructions. The final products were extracted from 2% agarose gel run in 1× TBE and purified with a GeneJET PCR Purification kit to allow for sample multiplexing during NGS. Barcoded PCR samples were pooled in equimolar amounts and submitted to the NGS Core Center, Department of Molecular Medicine, Aarhus University Hospital (Denmark) for (PairedEnd) Illumina Novaseq sequencing.

NGS data analysis

Raw data files (fastq.gz) were checked for data quality using fastqc.[35] From the quality control report, the necessary cleaning and pre-processing steps were taken before doing the downstream analysis (Figure S3). Adapter sequences were removed using Trimmomatic, and then, using the VSEARCH tool, forward and reverse reads were merged and converted to FASTA files using the fastq2fasta converter module for further processing.[36,37] Constant sequences were trimmed using custom Python scripts. The remaining aptamer sequences, along with their headers in the FASTA files, were used to generate count files using in-house scripts (written in Python) to obtain the number of each aptamer in each sample, which was used for further downstream analysis and visualization of the results. Enriched administration route-specific unique sequences were searched setting parameters to select the correct 36-nt-long sequences with enrichment of at least ≥10 sequence counts in cycle 10 or cycle 12. The complete code written with Javascript is available on GitHub: https://github.com/thejebo/RNA-Data-Processor/blob/master/index.js. The family search was based on ≥80% nucleotide conservation within the variable region. Generally, the motif search and sequence alignments were performed as described previously by Dupont et al.[6] The MEME suite was used for identifying conserved motifs among RNA sequences.[38,39] The following settings were used: number of sites (10–300), 0 or 1 motif occurrence per sequence, motif width (5–40), maximum number of different motifs (25), search given strand only. The significance level of the results was set to Expect value (E-value) of 0.05 depicting a 5% chance for an alignment to occur randomly. LocARNA was used for the sequence alignment with standard settings.[40]

Production of selected aptamers

The single-stranded DNA (ssDNA) template sequences (Sigma-Aldrich) for aptamer production were 5′-GGGGCCACCAACGACA TTGATTGTCCCAAATTATCCTTAGAACTTTTACCTCCAGTTG

ATATAAATAGTGCCCATGGATCCGCGGGTGTCGGG-3′ (Apt1), 5′-GGGGCCACCAACGACATTTTGACTGAATACGCACATTCGCCAAATTGCCGGCCCGTTGATATAAATAGTGCCCATGGATCCGCGGGTGTCGGG-3′ (Apt2), 5′-GGGGCCACCAACGACATTTGTACGCTCGCATTTGTGCGTTGGTGACCGCACTCAGTTGATATAAATAGTGCCCATGGATCCGCGGGTGTCGGG-3′ (Apt5). 5′-GGGGCCACCAACGACATTTACGTCGACTTAGTTGCGCCATGCTACAACCTAACGGTTGATATAAATAGTGCCCATGGATCCGCGGGTGTCGGG-3′ (Control apt), with similar constant regions used for control aptamer production. It is a sequence within the original aptamer pool but not detectable in the last sequenced SELEX cycles in the NGS pool. The aptamer production was performed as described on sections "Reverse transcription and PCR amplification" and "Purification of RNA with 8% polyacrylamide electrophoresis". The concentration of the starting ssDNA template was 2 μg.

In vivo biodistribution

A proof-of-concept *in vivo* biodistribution study was conducted for aptamers selected based on acquired NGS data. Animals, aptamer administration, tissue collection, RNA extraction, and reverse transcription to cDNA were performed similarly as presented on the section "Reverse transcription and PCR amplification". Each aptamer was administered intravenously at a dose of 1.2 nmol per rat.

The two-step RT-qPCR was performed using 4 ng synthesized cDNA, previously mentioned primers, PowerUp SYBR Green Master Mix (catalog no. A257, Thermo Fisher Scientific), and QuantStudio 5 Real-Time PCR System for Human Identification (catalog no. A34322, Thermo Fisher Scientific). A standard curve and positive and negative controls were included. Melt curve analysis was used in the optimization of the qPCR protocol. The data analysis was performed with QuantStudio Design & Analysis Software version 1.5.1, and the statistical analysis was performed with GraphPad Prism 10 for Windows 64-bit (version 10.0.2 (232)). Data were analyzed using a 2-way ANOVA with Tukey's multiple comparisons test. The level of statistical significance was set for $p < 0.05$. ΔCt was calculated as follows: $\Delta Ct = Ct_{Aptamer} - Ct_{Control\ apt}$, where Ct = cycle threshold value. The increase of 1 unit in the Ct value depicts the 2-fold difference in the original PCR template. All experiments were repeated at least 3 times. The data are presented as mean and SD.

Structural prediction and analysis of preferentially ocular targeting aptamers Apt2 and Apt5

The 3D structures of the aptamers were predicted with trRosettaRNA web server[41] and visualized with PyMOL. The CD spectra and melting temperature data were recorded using a Chirascan spectrometer (Applied Photophysics, Leatherhead, UK) equipped with a temperature controller (TC 125, Quantum Northwest, Liberty Lake, WA). The RNA aptamers were diluted with nf water and annealed in a water bath at 85°C for 2 min. Immediately after annealing, the aptamers were cooled on ice and diluted in a buffer containing 5 mM $MgCl2 \cdot 6H_2O$ (catalog no. 31413, Riedel-de Haën AG, Seelze, Germany) and 4× Dulbecco's PBS (DPBS 10× Liquid Concentrate, catalog no. 14200067, Thermo Fisher), with a final concentration of 15 μg/mL for Apt2 and Apt5. A quartz cuvette with a 1-mm path length was used for measurements. The CD spectra were recorded from 200 to 300 nm at 22°C using a scan speed of 0.5 s. After averaging 3 scans and subtracting the buffer baseline, each spectrum was processed with Savitzky-Golay smoothing using a window size of 11. The final CD spectra were represented as a unit of molar ellipticity:

$$[\theta] = \theta/(10 * d * c),$$

where θ represents measured ellipticity (millidegree), d the path length of the cuvette (cm), and c the concentration of RNA (mol/L). The melting temperature data were recorded from 22°C to 94°C in 2°C increments, with a temperature gradient of 1.0°C/min. The baseline corrected melting temperature data were recorded at 265 nm and fitted using Boltzmann's sigmoid equation:

$$[\theta] = [\theta]\mathrm{min} + ([\theta]\mathrm{max} - [\theta]\mathrm{min})/(1 + \exp((\mathrm{Tm} - \mathrm{T})\,/\,\mathrm{s}))$$

where $[\theta]$ represents the measured molar ellipticity at 265 nm, $[\theta]_{min}$ and $[\theta]_{max}$ the minimum and maximum values of $[\theta]$, T the temperature, T_m the melting point, and s the slope.

DATA AND CODE AVAILABILITY

Data will be made available on request.

ACKNOWLEDGMENTS

We acknowledge valuable guidance and help from Professor Arto Urtti and thank Professor Pasi Virta for his help and suggestions on the analysis of CD spectra. We thank Mia Pirskanen for her technical assistance. S.K. acknowledges financial support from the University of Eastern Finland, Sokeain Ystävät Foundation, and Instrumentarium Science Foundation. A.S. received funding for this study from the Research Council of Finland (333301) and the Instrumentarium Science Foundation.

AUTHOR CONTRIBUTIONS

S.K.: data curation, formal analysis, funding acquisition, investigation, methodology, validation, visualization, writing – original draft, and writing – review & editing. K.S.: data curation, formal analysis, investigation, methodology, and validation. U.S.: data curation, formal analysis, methodology, and writing – original draft. P.B.: methodology, visualization, and writing – review & editing. K.M.: investigation, data curation, visualization, and writing – review & editing. J.K.: conceptualization, resources, and writing – review & editing; D.M.D.: conceptualization, methodology, and writing – review & editing. A.S.: conceptualization, funding acquisition, methodology, resources, supervision, and writing – review & editing.

DECLARATION OF INTERESTS

A.S. has received financial support from Janssen Pharmaceutica. D.M.D. is a founder and shareholder of Aptamist ApS.

SUPPLEMENTAL INFORMATION

Supplemental information can be found online at https://doi.org/10.1016/j.omtn.2024.102352.

REFERENCES

1. Wong, W.L., Su, X., Li, X., Cheung, C.M.G., Klein, R., Cheng, C.-Y., and Wong, T.Y. (2014). Global prevalence of age-related macular degeneration and disease burden projection for 2020 and 2040: a systematic review and meta-analysis. Lancet Global Health *2*, e106–e116. https://doi.org/10.1016/S2214-109X(13)70145-1.

2. Purola, P.K.M., Nättinen, J.E., Ojamo, M.U.I., Koskinen, S.V.P., Rissanen, H.A., Sainio, P.R.J., and Uusitalo, H.M.T. (2021). Prevalence and 11-year incidence of common eye diseases and their relation to health-related quality of life, mental health, and visual impairment. Qual. Life Res. *30*, 2311–2327. https://doi.org/10.1007/s11136-021-02817-1.
3. Ammar, M.J., Hsu, J., Chiang, A., Ho, A.C., and Regillo, C.D. (2020). Age-related macular degeneration therapy: a review. Curr. Opin. Ophthalmol. *31*, 215–221. https://doi.org/10.1097/ICU.0000000000000657.
4. Subrizi, A., del Amo, E.M., Korzhikov-Vlakh, V., Tennikova, T., Ruponen, M., and Urtti, A. (2019). Design principles of ocular drug delivery systems: importance of drug payload, release rate, and material properties. Drug Discov. Today *24*, 1446–1457. https://doi.org/10.1016/j.drudis.2019.02.001.
5. del Amo, E.M., Rimpelä, A.-K., Heikkinen, E., Kari, O.K., Ramsay, E., Lajunen, T., Schmitt, M., Pelkonen, L., Bhattacharya, M., Richardson, D., et al. (2017). Pharmacokinetic aspects of retinal drug delivery. Prog. Retin. Eye Res. *57*, 134–185. https://doi.org/10.1016/j.preteyeres.2016.12.001.
6. Dupont, D.M., Larsen, N., Jensen, J.K., Andreasen, P.A., and Kjems, J. (2015). Characterisation of aptamer–target interactions by branched selection and high-throughput sequencing of SELEX pools. Nucleic Acids Res. *43*, e139. https://doi.org/10.1093/nar/gkv700.
7. Bjerregaard, N., Andreasen, P.A., and Dupont, D.M. (2016). Expected and unexpected features of protein-binding RNA aptamers. WIREs RNA *7*, 744–757. https://doi.org/10.1002/wrna.1360.
8. Kovacevic, K.D., Gilbert, J.C., and Jilma, B. (2018). Pharmacokinetics, pharmacodynamics and safety of aptamers. Adv. Drug Deliv. Rev. *134*, 36–50. https://doi.org/10.1016/j.addr.2018.10.008.
9. Kourlas, H., and Schiller, D.S. (2006). Pegaptanib sodium for the treatment of neovascular age-related macular degeneration: A review. Clin. Therapeut. *28*, 36–44. https://doi.org/10.1016/j.clinthera.2006.01.009.
10. Kang, C. (2023). Avacincaptad Pegol: First Approval. Drugs *83*, 1447–1453. https://doi.org/10.1007/s40265-023-01948-8.
11. Tuerk, C., and Gold, L. (1990). Systematic Evolution of Ligands by Exponential Enrichment: RNA Ligands to Bacteriophage T4 DNA Polymerase. Science *249*, 505–510. https://doi.org/10.1126/science.2200121.
12. Ellington, A.D., and Szostak, J.W. (1990). In vitro selection of RNA molecules that bind specific ligands. Nature *346*, 818–822. https://doi.org/10.1038/346818a0.
13. Sola, M., Menon, A.P., Moreno, B., Meraviglia-Crivelli, D., Soldevilla, M.M., Cartón-García, F., and Pastor, F. (2020). Aptamers Against Live Targets: Is In Vivo SELEX Finally Coming to the Edge? Mol. Ther. Nucleic Acids *21*, 192–204. https://doi.org/10.1016/j.omtn.2020.05.025.
14. Cheng, C., Chen, Y.H., Lennox, K.A., Behlke, M.A., and Davidson, B.L. (2013). In vivo SELEX for Identification of Brain-penetrating Aptamers. Mol. Ther. Nucleic Acids *2*, e67. https://doi.org/10.1038/mtna.2012.59.
15. Díaz-Coránguez, M., Ramos, C., and Antonetti, D.A. (2017). The inner blood-retinal barrier: Cellular basis and development. Vis. Res. *139*, 123–137. https://doi.org/10.1016/j.visres.2017.05.009.
16. Mi, J., Liu, Y., Rabbani, Z.N., Yang, Z., Urban, J.H., Sullenger, B.A., and Clary, B.M. (2010). In vivo selection of tumor-targeting RNA motifs. Nat. Chem. Biol. *6*, 22–24. https://doi.org/10.1038/nchembio.277.
17. Chen, L., He, W., Jiang, H., Wu, L., Xiong, W., Li, B., Zhou, Z., and Qian, Y. (2019). In vivo SELEX of bone targeting aptamer in prostate cancer bone metastasis model. Int. J. Nanomedicine *14*, 149–159. https://doi.org/10.2147/IJN.S188003.
18. Schwartz, K., and Budenz, D. (2004). Current management of glaucoma. Curr. Opin. Ophthalmol. *15*, 119–126. https://doi.org/10.1097/00055735-200404000-00011.
19. Grzybowski, A., Told, R., Sacu, S., Bandello, F., Moisseiev, E., Loewenstein, A., and Schmidt-Erfurth, U.; Euretina Board (2018). 2018 Update on Intravitreal Injections: Euretina Expert Consensus Recommendations. Ophthalmologica *239*, 181–193. https://doi.org/10.1159/000486145.
20. Mehta, N.S., and Emami-Naeini, P. (2022). A Review of Systemic Biologics and Local Immunosuppressive Medications in Uveitis. JOVR *17*, 276–289. https://doi.org/10.18502/jovr.v17i2.10804.
21. Bill, A., Törnquist, P., and Alm, A. (1980). Permeability of the intraocular blood vessels. Trans. Ophthalmol. Soc. U K *100*, 332–336.
22. Weigand, J.E., Sanchez, M., Gunnesch, E.-B., Zeiher, S., Schroeder, R., and Suess, B. (2008). Screening for engineered neomycin riboswitches that control translation initiation. RNA *14*, 89–97. https://doi.org/10.1261/rna.772408.
23. Kohlberger, M., and Gadermaier, G. (2022). SELEX: Critical factors and optimization strategies for successful aptamer selection. Biotechnol. Appl. Biochem. *69*, 1771–1792. https://doi.org/10.1002/bab.2244.
24. Jones, S., Daley, D.T., Luscombe, N.M., Berman, H.M., and Thornton, J.M. (2001). Protein-RNA interactions: a structural analysis. Nucleic Acids Res. *29*, 943–954. https://doi.org/10.1093/nar/29.4.943.
25. Ozer, A., Pagano, J.M., and Lis, J.T. (2014). New Technologies Provide Quantum Changes in the Scale, Speed, and Success of SELEX Methods and Aptamer Characterization. Mol. Ther. Nucleic Acids *3*, e183. https://doi.org/10.1038/mtna.2014.34.
26. Levay, A., Brenneman, R., Hoinka, J., Sant, D., Cardone, M., Trinchieri, G., Przytycka, T.M., and Berezhnoy, A. (2015). Identifying high-affinity aptamer ligands with defined cross-reactivity using high-throughput guided systematic evolution of ligands by exponential enrichment. Nucleic Acids Res. *43*, e82. https://doi.org/10.1093/nar/gkv534.
27. Lincoff, A.M., Mehran, R., Povsic, T.J., Zelenkofske, S.L., Huang, Z., Armstrong, P.W., Steg, P.G., Bode, C., Cohen, M.G., Buller, C., et al. (2016). Effect of the REG1 anticoagulation system versus bivalirudin on outcomes after percutaneous coronary intervention (REGULATE-PCI): a randomised clinical trial. Lancet *387*, 349–356. https://doi.org/10.1016/S0140-6736(15)00515-2.
28. Ganson, N.J., Povsic, T.J., Sullenger, B.A., Alexander, J.H., Zelenkofske, S.L., Sailstad, J.M., Rusconi, C.P., and Hershfield, M.S. (2016). Pre-existing anti–polyethylene glycol antibody linked to first-exposure allergic reactions to pegnivacogin, a PEGylated RNA aptamer. J. Allergy Clin. Immunol. *137*, 1610–1613.e7. https://doi.org/10.1016/j.jaci.2015.10.034.
29. Lee, C.H., Lee, S.-H., Kim, J.H., Noh, Y.-H., Noh, G.-J., and Lee, S.-W. (2015). Pharmacokinetics of a Cholesterol-conjugated Aptamer Against the Hepatitis C Virus (HCV) NS5B Protein. Mol. Ther. Nucleic Acids *4*, e254. https://doi.org/10.1038/mtna.2015.30.
30. Hirota, M., Murakami, I., Ishikawa, Y., Suzuki, T., Sumida, S.i., Ibaragi, S., Kasai, H., Horai, N., Drolet, D.W., Gupta, S., et al. (2016). Chemically Modified Interleukin-6 Aptamer Inhibits Development of Collagen-Induced Arthritis in Cynomolgus Monkeys. Nucleic Acid Therapeut. *26*, 10–19. https://doi.org/10.1089/nat.2015.0567.
31. Rosenberg, J.E., Bambury, R.M., Van Allen, E.M., Drabkin, H.A., Lara, P.N., Harzstark, A.L., Wagle, N., Figlin, R.A., Smith, G.W., Garraway, L.A., et al. (2014). A phase II trial of AS1411 (a novel nucleolin-targeted DNA aptamer) in metastatic renal cell carcinoma. Invest. N. Drugs *32*, 178–187. https://doi.org/10.1007/s10637-013-0045-6.
32. Fjelstrup, S., Dupont, D.M., Bus, C., Enghild, J.J., Jensen, J.B., Birkenkamp-Demtröder, K., Dyrskjøt, L., and Kjems, J. (2022). Differential RNA aptamer affinity profiling on plasma as a potential diagnostic tool for bladder cancer. NAR Cancer *4*, zcac025. https://doi.org/10.1093/narcan/zcac025.
33. Madsen, J.B., Dupont, D.M., Andersen, T.B., Nielsen, A.F., Sang, L., Brix, D.M., Jensen, J.K., Broos, T., Hendrickx, M.L.V., Christensen, A., et al. (2010). RNA Aptamers as Conformational Probes and Regulatory Agents for Plasminogen Activator Inhibitor-1. Biochemistry *49*, 4103–4115. https://doi.org/10.1021/bi100066j.
34. Krissanaprasit, A., Key, C.M., Froehlich, K., Pontula, S., Mihalko, E., Dupont, D.M., Andersen, E.S., Kjems, J., Brown, A.C., and LaBean, T.H. (2021). Multivalent Aptamer-Functionalized Single-Strand RNA Origami as Effective, Target-Specific Anticoagulants with Corresponding Reversal Agents. Adv. Healthcare Mater. *10*, e2001826. https://doi.org/10.1002/adhm.202001826.
35. Wingett, S.W., and Andrews, S. (2018). FastQ Screen: A tool for multi-genome mapping and quality control. F1000Res. *7*, 1338. https://doi.org/10.12688/f1000research.15931.2.
36. Bolger, A.M., Lohse, M., and Usadel, B. (2014). Trimmomatic: a flexible trimmer for Illumina sequence data. Bioinformatics *30*, 2114–2120. https://doi.org/10.1093/bioinformatics/btu170.

37. Rognes, T., Flouri, T., Nichols, B., Quince, C., and Mahé, F. (2016). VSEARCH: a versatile open source tool for metagenomics. PeerJ *4*, e2584. https://doi.org/10.7717/peerj.2584.

38. Bailey, T.L., and Elkan, C. (1994). Fitting a mixture model by expectation maximization to discover motifs in biopolymers. Proc. Int. Conf. Intell. Syst. Mol. Biol. *2*, 28–36.

39. Bailey, T.L., Boden, M., Buske, F.A., Frith, M., Grant, C.E., Clementi, L., Ren, J., Li, W.W., and Noble, W.S. (2009). MEME SUITE: tools for motif discovery and searching. Nucleic Acids Res. *37*, W202–W208. https://doi.org/10.1093/nar/gkp335.

40. Smith, C., Heyne, S., Richter, A.S., Will, S., and Backofen, R. (2010). Freiburg RNA Tools: a web server integrating INTARNA, EXPARNA and LOCARNA. Nucleic Acids Res. *38*, W373–W377. https://doi.org/10.1093/nar/gkq316.

41. Wang, W., Feng, C., Han, R., Wang, Z., Ye, L., Du, Z., Wei, H., Zhang, F., Peng, Z., and Yang, J. (2023). trRosettaRNA: automated prediction of RNA 3D structure with transformer network. Nat. Commun. *14*, 7266. https://doi.org/10.1038/s41467-023-42528-4.

OMTN, Volume *35*

Supplemental information

Targeting ocular tissues with intravenously administered aptamers selected by *in vivo* SELEX

Sonja Korhonen, Katja Stenberg, Umair Seemab, Piia Bartos, Katariina Mäkiniemi, Jørgen Kjems, Daniel Miotto Dupont, and Astrid Subrizi

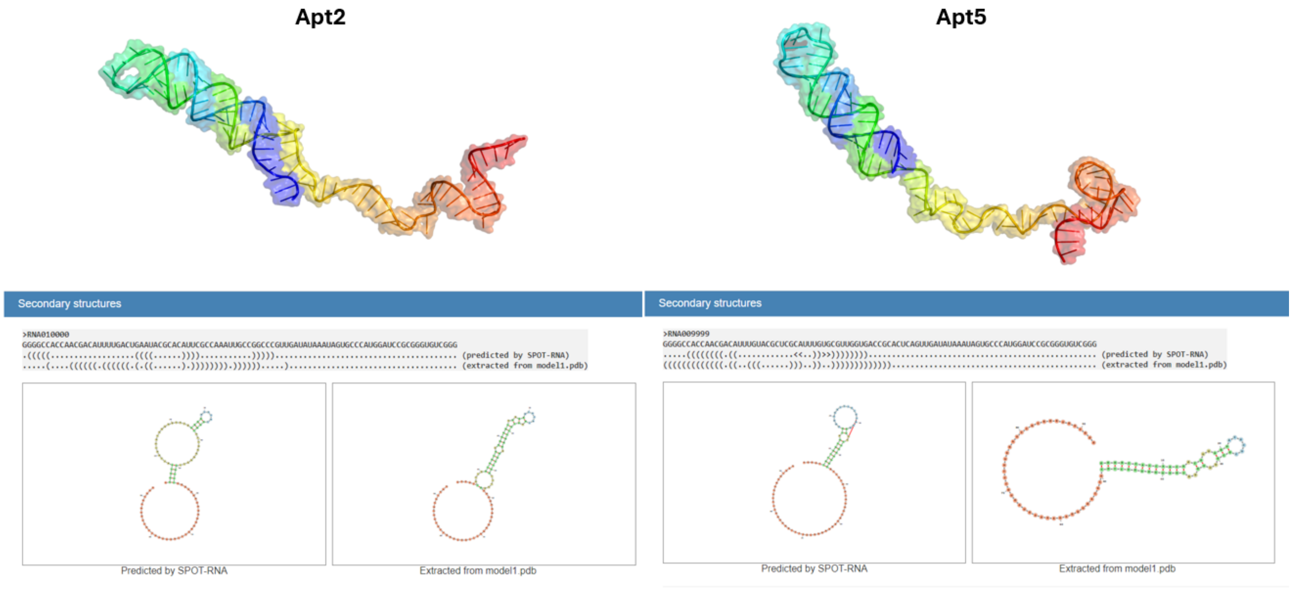

Figure S1: Secondary structures and 3D models for Apt2 and Apt5 built based on de novo folding guided by deep learning restraints with the trRosettaRNA.

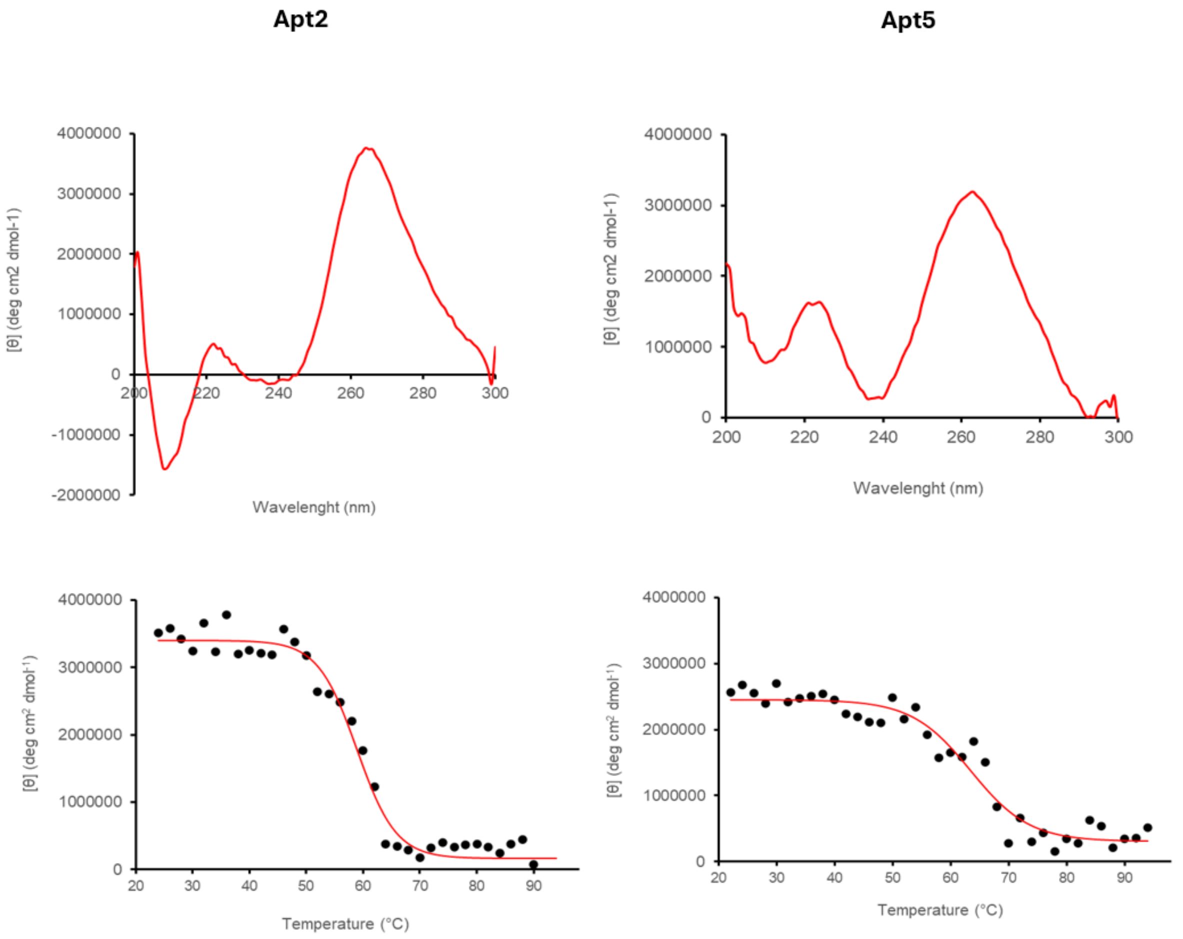

Figure S2. Characterization of the Aptamers Apt2 and Apt5 using circular dichroism (CD) and melting temperature analysis. The final spectra were represented as a unit of molar ellipticity. The top row represents the smoothed CD spectra of Apt2 and Apt5 and the lower row melting curves used for melting temperature detection.

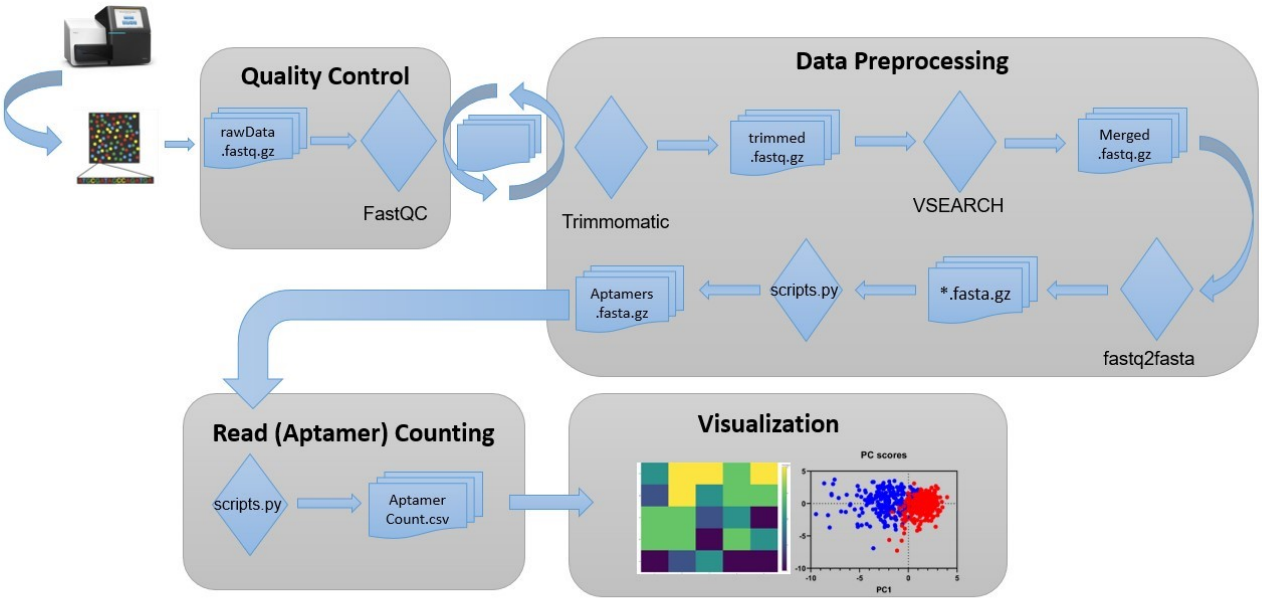

Figure S3: Aptamer data analysis workflow from raw data files to read count in each sample for further downstream analysis.

Table S1: Percentage of the 50 most enriched 36 nucleotide sequences of total sequence count pooled from SELEX cycles 10, 12, and 14. Only the 36 nucleotide-long variable regions are shown.

Aptamer sequence	Percentage of the pool (%)
GATTGTCCCAAATTATCCTTAGAACTTTTACCTCCA	34.53
TTGACTGAATACGCACATTCGCCAAATTGCCGGCCC	3.89
ATGCCGTCCGACCCACTCGTACGGCACTATCTCCCC	3.48
CAATGCCCTCATGTTTTTGCTCAAAACATCACTGCT	2.33
TGTACGCTCGCATTTGTGCGTTGGTGACCGCACTCA	0.73
GATTGTCCCAAATTATCCTTAGAACTTTTATCTCCA	0.67
TCTGCTAGGCGGGATTTTCGTCAGTGTCCCACCCAT	0.54
ACAAACCAAGAGTCGCTATTGTCGGTTGATGCTCCC	0.54
GGTAGCACCCGCCAATCGACTTTTCACCGCAGAAGC	0.53
ATGCCGTCCGACCCACTCGTACGGCACTAACTCCCC	0.43
GATTGTCCCAAACTATCCTTAGAACTTTTACCTCCA	0.28
GTTGCCCTTCAAACTGACGACCGACCGTCGTGAAAA	0.24
GGTGTCCTGCCATATAACTAGTTACCTCGACCTATC	0.23
GTCGCACCATCGAGCGCGCACAATAGCGTGTCCCCC	0.22
GATTGCCCCAAATTATCCTTAGAACTTTTACCTCCA	0.19

GGATGGGCTCCTTCGCAACGATGTACCGCATACGAC	0.19
ATGCCGTCCGATCCACTCGTACGGCACTATCTCCCC	0.16
GGTTCGTACCCTCTATGACATCACGGCTCGCGCGAT	0.14
CTACTCAACCGGTTTGATCTAGATTGCTCGGCCCCA	0.14
GTTGCTGACCAGTTGCGTTACGACATCACTCTCGAT	0.14
GATCGTCCCAAATTATCCTTAGAACTTTTACCTCCA	0.14
GAATGAACATCCAGTCTTTGTCTTTCCTTGCTCCCC	0.13
TTTCGCAGTCGATATCTTCTTTCGTTGGCACCCCAA	0.13
GGTTTCGCCAATCACTACAACGTACGTGCCCGTATG	0.13
CCGGTGAGCCTTTCCGCACGCTTGAGCAAGTCACGC	0.13
TGTTGTCTGGATTTGCCGATAGTCACGTTACGTAAC	0.12
ACAATGTCACTTAAATCCCGGCCCGAATCACGTAAC	0.12
CGTATGTCTGACCCTGACATAAAACGTTAGCGTAAC	0.12
TGGTTAAGAGCAACTGCCCTAATCTCACAATGCCCC	0.11
GGTTGCCTGCATTTACGCTGTTTACGCCTTCGCAGC	0.11
CGTGCCGACCCGCATGTCCACGGTGCTCCACACACC	0.11
GGTCTGCCTTTTCATATAACCGTTTATCTCACTCCC	0.11
GATTGTCCCAAATCATCCTTAGAACTTTTACCTCCA	0.10
TACTTACGTTTATGTCCTGCGGTCTACGCCCTCCCC	0.10
GTTTGGCTTCCATCCGCGTATCAACCGTCGACCCGT	0.10
CGCGCTCGTTAGCAATTTCGGTGTCCCGTTATAATC	0.10
GGCGAGCCGCAGCACCGACTTCACGCATACGCGTTC	0.10
CTCGGAAAACCGCTGATTTCGCTCACTGACCCCACA	0.09
GTTACAAAATGTCCCCGCATGCGTCCACGCTCTCGC	0.09
GAGTTGCCCTTATCCTCCCAACGCATGCACCAGCGC	0.08
GGAATGCCCGCCTCTAACGCTCAGGCCGTCTGCTGC	0.08
CACCCCAACGCCCCTTGCGGACAAGCGTTGTCCTGC	0.08
TGTTCCCTTATTGTAAACACACGTGCCCTCGTGTGC	0.08
CGCCCATATCACTGGGACTTGACGTATCTTGGCCGC	0.08
CGGCTGCCAACCGCATATCGTGAAGGCTGGCTTCGA	0.08
GATTGTCCCAAATTATCCTTAGAACCTTTACCTCCA	0.08
TCTTGCCGAGTGCGCATCGACAAGTCAGAATGCCCC	0.07
GATTGTCCCAAATTATCCTTAGAACTTTTACCTCAA	0.07
CGTACAGACGTGCACGCCAAACGTCGTACGGCCTCG	0.07
CGCACGCAACGCCGCTGTTCCGTGAGATTGAGCCCT	0.07

Table S2: Change in the percentage of the 50 most enriched 36 nucleotide sequences of total sequence count from SELEX cycles 10, 12, and 14. Only the 36 nucleotide-long variable regions are shown.

Aptamer sequence	Percentage of the pool (%)		
	Cycle 10	Cycle 12	Cycle 14

GATTGTCCCAAATTATCCTTAGAACTTTTACCTCCA	26,10	40,05	38,83
TTGACTGAATACGCACATTCGCCAAATTGCCGGCCC	1,31	4,16	5,27
ATGCCGTCCGACCCACTCGTACGGCACTATCTCCCC	4,70	5,12	2,39
CAATGCCCTCATGTTTTTGCTCAAAACATCACTGCT	2,40	3,78	1,67
TGTACGCTCGCATTTGTGCGTTGGTGACCGCACTCA	0,03	0,10	1,51
GATTGTCCCAAATTATCCTTAGAACTTTTATCTCCA	0,29	0,74	0,86
TCTGCTAGGCGGGATTTTCGTCAGTGTCCCACCCAT	0,02	0,22	1,03
ACAAACCAAGAGTCGCTATTGTCGGTTGATGCTCCC	0,39	0,58	0,65
GGTAGCACCCGCCAATCGACTTTTCACCGCAGAAGC	0,52	0,73	0,46
ATGCCGTCCGACCCACTCGTACGGCACTAACTCCCC	0,14	0,41	0,62
GATTGTCCCAAACTATCCTTAGAACTTTTACCTCCA	0,16	0,30	0,35
GTTGCCCTTCAAACTGACGACCGACCGTCGTGAAAA	0,15	0,29	0,26
GGTGTCCTGCCATATAACTAGTTACCTCGACCTATC	0,01	0,06	0,46
GTCGCACCATCGAGCGCGCACAATAGCGTGTCCCCC	0,06	0,15	0,36
GATTGCCCCAAATTATCCTTAGAACTTTTACCTCCA	0,15	0,24	0,21
GGATGGGCTCCTTCGCAACGATGTACCGCATACGAC	0,26	0,25	0,14
ATGCCGTCCGATCCACTCGTACGGCACTATCTCCCC	0,21	0,25	0,11
GGTTCGTACCCTCTATGACATCACGGCTCGCGCGAT	0,29	0,17	0,08
CTACTCAACCGGTTTGATCTAGATTGCTCGGCCCCA	0,16	0,23	0,10
GTTGCTGACCAGTTGCGTTACGACATCACTCTCGAT	0,16	0,07	0,20
GATCGTCCCAAATTATCCTTAGAACTTTTACCTCCA	0,07	0,16	0,18
GAATGAACATCCAGTCTTTGTCTTTCCTTGCTCCCC	0,16	0,16	0,13
TTTCGCAGTCGATATCTTCTTTCGTTGGCACCCCAA	0,02	0,10	0,22
GGTTTCGCCAATCACTACAACGTACGTGCCCGTATG	0,14	0,07	0,17
CCGGTGAGCCTTTCCGCACGCTTGAGCAAGTCACGC	0,18	0,17	0,09
TGTTGTCTGGATTTGCCGATAGTCACGTTACGTAAC	0,12	0,05	0,19
ACAATGTCACTTAAATCCCGGCCCGAATCACGTAAC	0,26	0,16	0,05
CGTATGTCTGACCCTGACATAAAACGTTAGCGTAAC	0,26	0,15	0,05
TGGTTAAGAGCAACTGCCCTAATCTCACAATGCCCC	0,01	0,04	0,22
GGTTGCCTGCATTTACGCTGTTTACGCCTTCGCAGC	0,07	0,07	0,17
CGTGCCGACCCGCATGTCCACGGTGCTCCACACACC	0,21	0,13	0,06
GGTCTGCCTTTTCATATAACCGTTTATCTCACTCCC	0,01	0,08	0,17
GATTGTCCCAAATCATCCTTAGAACTTTTACCTCCA	0,08	0,12	0,12
TACTTACGTTTATGTCCTGCGGTCTACGCCCTCCCC	0,21	0,14	0,04
GTTTGGCTTCCATCCGCGTATCAACCGTCGACCCGT	0,01	0,06	0,17
CGCGCTCGTTAGCAATTTCGGTGTCCCGTTATAATC	0,02	0,09	0,14
GGCGAGCCGCAGCACCGACTTCACGCATACGCGTTC	0,36	0,07	0,02
CTCGGAAAACCGCTGATTTCGCTCACTGACCCCACA	0,07	0,11	0,09
GTTACAAAATGTCCCCGCATGCGTCCACGCTCTCGC	0,14	0,06	0,09
GAGTTGCCCTTATCCTCCCAACGCATGCACCAGCGC	0,13	0,07	0,09
GGAATGCCCGCCTCTAACGCTCAGGCCGTCTGCTGC	0,01	0,06	0,14
CACCCCAACGCCCCTTGCGGACAAGCGTTGTCCTGC	0,05	0,14	0,07
TGTTCCCTTATTGTAAACACACGTGCCCTCGTGTGC	0,10	0,03	0,12
CGCCCATATCACTGGGACTTGACGTATCTTGGCCGC	0,05	0,07	0,11
CGGCTGCCAACCGCATATCGTGAAGGCTGGCTTCGA	0,11	0,08	0,07

GATTGTCCCAAATTATCCTTAGAACCTTTACCTCCA	0,04	0,08	0,10
TCTTGCCGAGTGCGCATCGACAAGTCAGAATGCCCC	0,01	0,03	0,14
GATTGTCCCAAATTATCCTTAGAACTTTTACCTCAA	0,03	0,08	0,10
CGTACAGACGTGCACGCCAAACGTCGTACGGCCTCG	0,01	0,04	0,12
CGCACGCAACGCCGCTGTTCCGTGAGATTGAGCCCT	0,01	0,07	0,10
